# IGFBP5 derived from endothelial cells drive vascular invasion and liver metastasis of colorectal cancer by activating the ITGB3/PI3K/AKT Axis

**DOI:** 10.1038/s41419-026-09081-6

**Published:** 2026-07-23

**Authors:** Nuersimanguli Maimaitiming, Yiqiao Deng, Huayuan Liu, Junshuai Xue, Chengyao Guo, Xiaomeng Liu, Bingzhi Wang, Xiao Chen, Zhen Huang, Hong Zhao, Jianqiang Cai

**Affiliations:** 1 Department of Hepatobiliary Surgery, State Key Laboratory of Molecular Oncology, National Cancer Center/National Clinical Research Center for Cancer/Cancer Hospital, Chinese Academy of Medical Sciences and Peking Union Medical College, Beijing, China; 2 Department of Pathology, State Key Laboratory of Molecular Oncology, National Cancer Center/National Clinical Research Center for Cancer/Cancer Hospital, Chinese Academy of Medical Sciences and Peking Union Medical College, Beijing, China

**Keywords:** Cancer, Metastasis

## Abstract

Vascular invasion (VascI) is a critical step in colorectal cancer (CRC) metastasis, but how specific cells in the tumor microenvironment (TME) regulate this process remains unclear, particularly the role of endothelial cells (ECs). Here, we found that vascular invasion is strongly associated with distant metastasis and poor prognosis in CRC patients. Analysis of single-cell RNA sequencing (scRNA-seq) data from 8 colorectal cancer liver metastases (CRLMs), included 4 VascI^+^ CRLMs and 4 VascI^-^ CRLMs, and identified 10 distinct EC subpopulations. Notably, an IGFBP5_tipECs significantly enriched in VascI^+^ CRLMs. High IGFBP5 expression correlated with VascI^+^ status and poor patient prognosis. Functionally, IGFBP5 overexpression (OE) in human umbilical vein endothelial cells (HUVECs) enhanced EC migration, tube formation, barrier disruption, and endothelial-to-mesenchymal transition (EndMT). In co-culture system, IGFBP5-OE ECs promoted malignant epithelial cells (MCs) proliferation, migration, and adhesion, and accelerated the growth of patient-derived CRC organoids. Mechanistically, RNA sequencing nominated ITGB3 as a key downstream effector, with activation of the PI3K/AKT signaling pathway. We confirmed a direct ligand-receptor interaction between EC-derived IGFBP5 and ITGB3 on MCs using multiplex immunofluorescence (mIF), co-immunoprecipitation (Co-IP), and proximity ligation assays (PLA). This interaction activated PI3K/AKT signaling in MCs, driving aggressive behavior both in vitro and in vivo, an effect that was significantly attenuated by ITGB3 silencing in MCs. Our findings identify IGFBP5-OE ECs as a key driver of VascI and liver metastasis in CRC via the ITGB3/PI3K/AKT axis, highlighting a potential therapeutic target to suppress metastatic progression.

**Figure:**
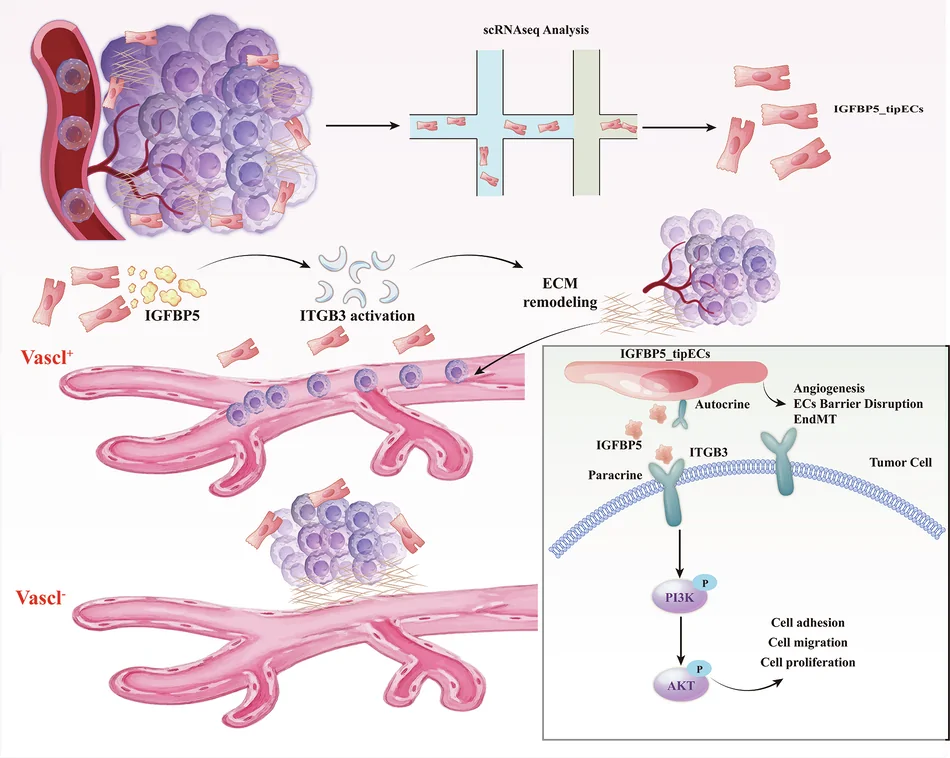

## Introduction

Colorectal cancer (CRC) is the second most common cause of cancer-related mortality worldwide, with an annual incidence increase of 1–2% among adults under 55 years of age [1]. CRC has a strong propensity for distant metastasis, particularly to the liver; over 50% of patients develop liver metastases (LM) during the course of their disease, which is associated with a markedly reduced 5-year survival rate [2, 3]. The treatment of colorectal cancer liver metastases (CRLM) involves multiple modalities, including liver resection, chemotherapy, radiotherapy, hepatic artery embolization, ablation, and immunotherapy [4, 5]. However, only a few patients benefit from these interventions. Owing to tumor heterogeneity and variations in treatment response, over 70% of patients experience disease progression [6]. Therefore, managing CRLM remains a major challenge. We propose that risk stratification based on distinct clinical features and subsequent exploration of the underlying mechanisms for each subgroup represent a promising strategy for developing more effective, personalized therapies.

Lymphovascular invasion (LVI) is an independent risk factor for LM and CRC prognosis [7, 8]. Pathologically, LVI signifies the presence of tumor cells within vascular or lymphatic channels, representing a critical step in the metastatic cascade [9]. Although LVI encompasses both lymphatic and vascular invasion (VascI), these two processes are driven by distinct mechanisms [10, 11]. Because CRLM occurs primarily via the hematogenous route, a precise understanding of VascI is essential, as it constitutes the most direct pathogenic pathway to the liver [6]. The intravasation of cancer cells into the vascular system and their colonization within vessels to form tumor-associated VascI appear to involve complex interactions between cancer cells and perivascular stromal cells [12]. However, the cellular signature and precise components that contribute to VascI formation in CRLM remain underexplored.

As the structural lining of blood vessels, endothelial cells (ECs) are the primary interface encountered by circulating tumor cells. In the tumor microenvironment (TME), activated cancer-associated ECs promote angiogenesis, resulting in a vasculature that is structurally and functionally abnormal [13–15]. This dysfunction is characterized by hypoxia, acidosis, and elevated interstitial pressure, impairing blood flow and facilitating tumor cell adhesion to the vessel wall, which is the initial step in VascI that leads to hematogenous LM [16–18]. Although the roles of ECs in promoting vessel sprouting (e.g., through tip/stalk cell dynamics) are well established, their specific functional contributions to VascI formation remain largely elusive [19, 20]. Therefore, a precise understanding of the mechanisms by which ECs regulate this process is crucial.

To elucidate the mechanisms underlying VascI and its role in driving LM, we used single-cell RNA sequencing (scRNA-seq) to characterize the heterogeneous landscape of ECs in patients with CRLM and VascI. The analysis identified a specific EC subpopulation marked by high IGFBP5 expression, suggesting its role in this process. We further validated the critical role of IGFBP5 in ECs and tumor cells through experiments at the cellular, patient derived CRC organoids, and tissue levels. Moreover, molecular docking, multicolor immunofluorescence (mIF) and spatial transcriptomics analysis demonstrated that IGFBP5^+^ ECs promote VascI and CRLM by activating ITGB3 on the surface of tumor cells. Our findings provide a comprehensive characterization of EC subgroups contributing to the VascI^+^ TME and suggest that targeting IGFBP5 over-expressed ECs is a promising strategy for stratified treatment of patients with CRLM and VascI.

## Results

### Single-cell profiling reveals VascI^+^ microenvironmental remodeling in CRLM

VascI is considered as a key factor associated with poor patient prognosis. To further confirm its role in CRC, bulk transcriptomic data from patients with CRC (including colorectal and rectal adenocarcinoma) were obtained from The Cancer Genome Atlas (TCGA) database. Using this dataset, we identified a cohort of patients with annotated VascI status, comprising 63 VascI^+^ and 166 VascI^-^ cases. Kaplan-Meier survival analysis showed that the overall survival (OS), progression free interval (PFI), and disease-specific survival (DSS) were significantly lower in VascI^+^ patients than in VascI^-^ patients (OS: hazard ratio [HR], 2.26; 95% confidence interval [CI], 1.21–4.21; *P* = 0.011; PFI: HR, 2.57; 95% CI, 1.51–4.39; *P* < 0.001; DSS: HR, 2.92; 95% CI, 1.50–5.66; *P* = 0.002) (Fig. S1A–C**)**. These findings confirm that VascI status may be an important clinical indicator of poor prognosis in CRC.

**Fig. 1:**
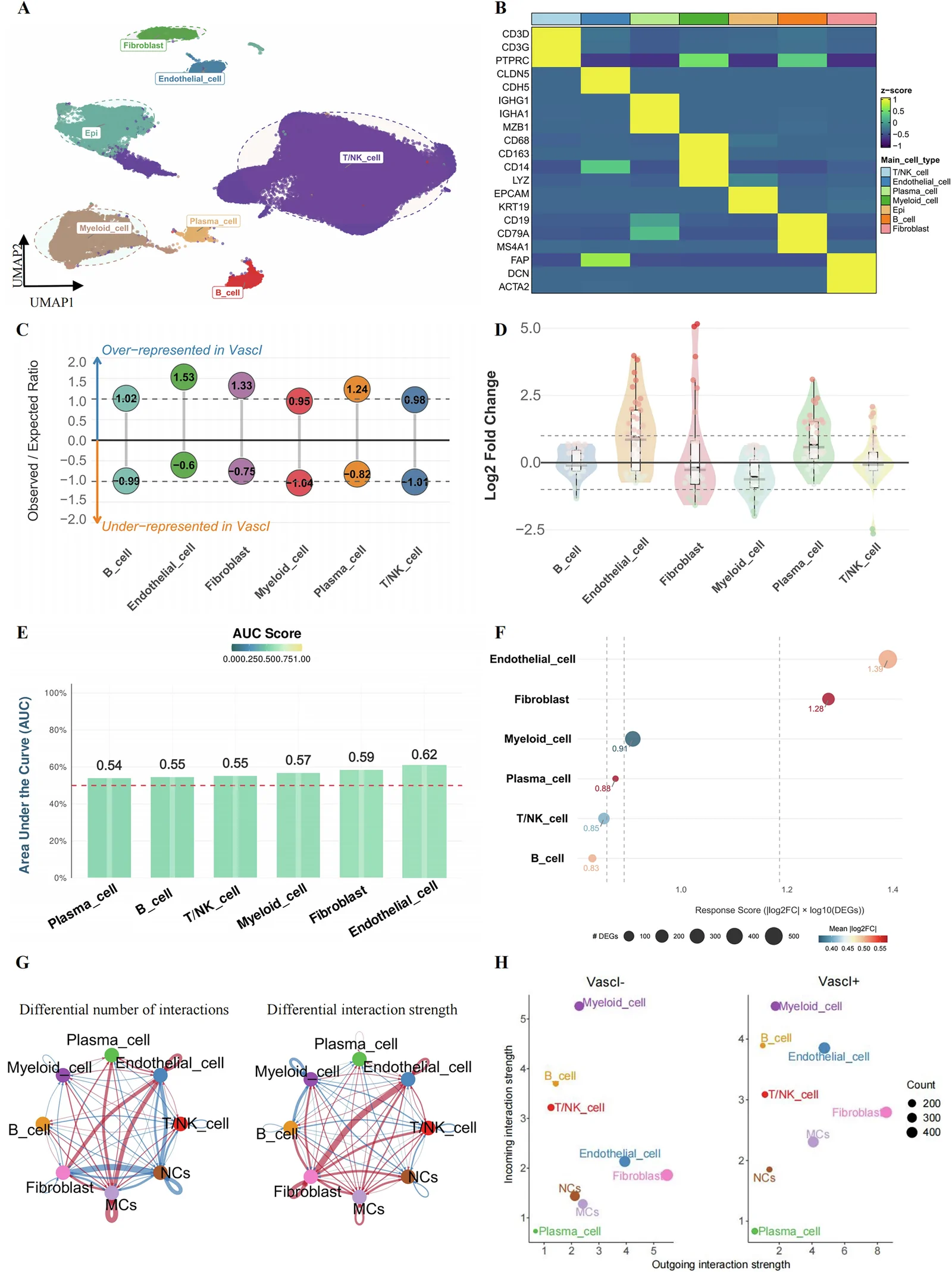
Single-Cell Profiling Reveals VascI^+^ Microenvironmental Remodeling in CRLM. **A** UMAP visualization of 8 sample and 95,206 high-quality cells clustered into 7 distinct subsets each represented by a different color. **B** Heatmap displaying the expression of canonical marker genes for each cell subsets. **C** Relative abundance of each cell lineage in VascI^+^ LM versus VascI^-^ LM samples, analyzed by R/oe. **D** MiloR differential abundance testing, with red points indicating neighborhoods significantly enriched in VascI^+^ LM. **E** Augur analysis ranking cell type responsiveness to VascI status based on multivariate AUC. **F** Differential gene variability analysis quantifying transcriptome reprogramming for each cell type. **G** Circle plots from CellChat analysis depicting ligand-receptor interactions between MCs and other populations in VascI^+^ LM and VascI^-^ LM. **H** Two-dimensional micro-environmental profiling showing incoming and outgoing interaction strength of each cell population.

To elucidate the TME characteristics of CRLM associated with VascI status, we conducted scRNA-seq analysis. The analytical workflow is illustrated in Supplementary Fig. 1D. Following stringent quality control (Supplementary Fig. 1E, F**)** and batch correction **(**Supplementary Fig. 1J, H), 95,206 high-quality cells were retained for downstream analysis. Unsupervised clustering identified seven major cell lineages **(**Fig. 1A**)**: T/NK cells (CD3D, CD3G, PTPRC), B cells (CD19, CD79A, MS4A1), plasma cells (IGHG1, IGHA1, MZB1), meloid cells (CD68, CD163, CD14, LYZ), epithelial cells (EPCAM, KRT19), fibroblasts (FAP, DCN, ACTA2), and ECs (CLDN5, CDH5). The validation of marker gene expression is shown in Fig. 1B.

Cell abundance analysis using R/oe and MiloR demonstrated preferential enrichment of ECs, fibroblasts, plasma cells, and B cells in VascI^+^ LM samples **(**Fig. 1C, D**)**, with ECs exhibiting the most pronounced increase. In contrast, VascI^-^ LM samples showed no significant distribution bias. Responsiveness analysis using the Augur algorithm **(**Fig. 1E**)** and intergroup differential gene variability assessment **(**Fig. 1F**)** consistently identified ECs as the cell population most responsive to VascI status, indicating profound transcriptome reprogramming.

Epithelial cells were stratified by copy number variation (CNV) analysis, using 500 T/NK, 500 plasma, and 500 B cells as reference populations. Extensive CNV alterations confirmed the presence of malignant epithelial cells (MCs) compared with normal epithelial cells **(**Supplementary Fig. 2A, B, C**)**. GSEA demonstrated significant upregulation of angiogenesis, cell cycle, and invasion pathways in VascI^+^ LM-derived MCs compared with the VascI^-^ LM counterparts **(**Supplementary Fig. 2D, E**)**.

Pseudo-three-dimensional spatial mapping using CSOmap **(**Supplementary Fig. 3A–D**)** revealed enhanced MCs interactions with fibroblasts, ECs, myeloid cells, B cells, and plasma cells in VascI^+^ LM **(**Supplementary Fig. 3E**)** compared with VascI^-^ LM **(**Supplementary Fig. 3F**)**. CellChat analysis **(**Fig. 1G**)** corroborated these findings, showing increased communication strength and ligand-receptor pair number between MCs and other populations, particularly ECs. Two-dimensional microenvironmental profiling further highlighted the intensified signal sender and receiver roles of ECs in VascI^+^ LM **(**Fig. 1H**)**. These results showed that VascI^+^ is associated with CRLM and contributes to the remodeling of its microenvironment.

### Construction of a VascI associated ECs atlas in CRLM

**Fig. 2:**
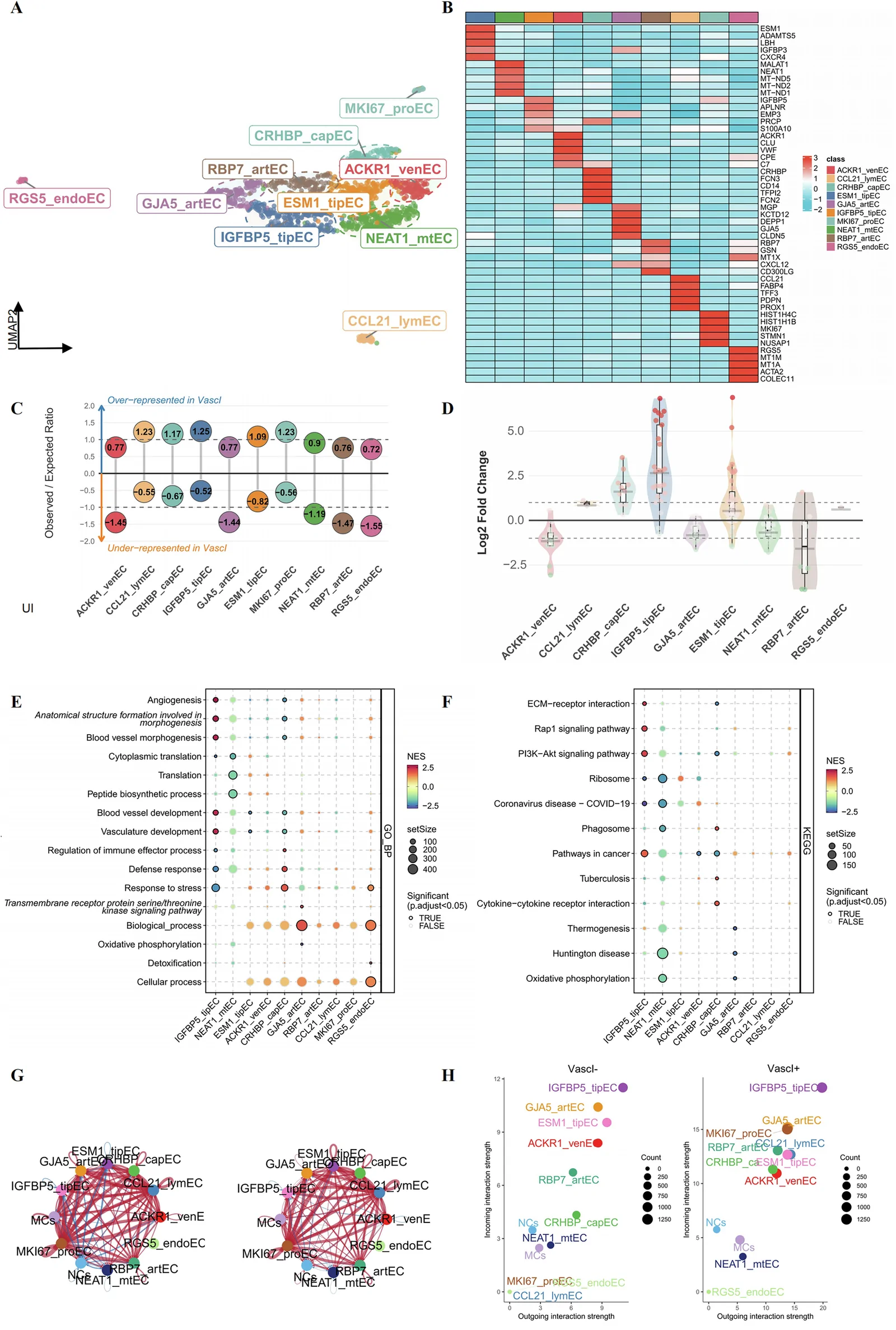
IGFBP5_tipEC are associated with VascI and CRLM. **A** UMAP visualization of endothelial cell (EC) subclustering. UMAP visualization of EC subclustering, identifying 10 distinct EC subsets. **B** Heatmap showing the expression of canonical marker genes for each EC subset. **C** Relative abundance of EC subsets in VascI^+^ LM versus VascI^-^ LM samples, analyzed by R/oe. **D** MiloR differential abundance testing of EC subsets between VascI^+^ LM and VascI^-^ LM conditions. **E** GO enrichment analysis showing distinct functional profiles for each EC subset. **F** KEGG pathway analysis comparing functional activities across EC subsets. **G** Circle plots from CellChat analysis depicting interaction patterns between malignant cells (MCs) and EC subsets in VascI^+^ LM (left) and VascI^-^ LM (right) microenvironments. **H** Two-dimensional micro-environmental profiling showing the outgoing and incoming interaction strength of each EC subset.

ECs contribute to VascI in various solid tumors, yet their role in CRLM remains unclear [21–23]. Given the high heterogeneity of ECs and our discovery that EC is the primary cell type associated with VascI, we will now attempt to identify which specific EC subgroups may drive VascI and liver metastasis. Based on our earlier findings, we focused on the EC subsets associated with VascI progression in CRLM. UMAP visualization of ECs analysis identified different EC subgroups, including ACKR1_venous ECs (venECs), CCL21_ lymphatic ECs (lymECs), CRHBP_capillary ECs (capECs), ESM1_tip ECs (tipECs), GJA5_arterial ECs (artECs), IGFBP5_tipECs (named because IGFBP5 is its top differentially expressed gene and encodes a secreted effector; importantly, and the functional role of IGFBP5 is explored below), MKI67_proliferating ECs (proECs), NEAT1_ mitochondrial-enriched ECs (mtECs), RBP7_artECs, and RGS5_endothelial-to-mesenchymal transition ECs (endoECs) **(**Fig. 2A, B**)**.

Comparative analyses using the Ro/e and MiloR algorithms revealed that CCL21_ lymECs, CRHBP_capECs, IGFBP5_tipECs, and MKI67_proECs were enriched in VascI^+^ LM samples, whereas ACKR1_venECs, GJA5_ artECs, NEAT1_mtECs, RBP7_artECs, and RGS5_endoECs were more prevalent in VascI^+^ LM **(**Fig. 2C, D**)**. Gene Ontology (GO) enrichment analysis revealed significant differences in functional enrichment across EC subpopulations **(**Fig. 2E**)**, showing distinct functional profiles for each EC subtype. Specifically, IGFBP5_tipEC expression was positively associated with angiogenesis and vascular developmental processes but negatively correlated with the cellular response to stress. In contrast, CRHBP_capECs showed an enrichment pattern opposite to that of IGFBP5 _tipECs. NEAT1_mtECs were negatively associated with cytoplasmic translation and peptide biosynthesis, whereas GJA5_artECs and RGS5_endoECs were enriched in broader biological processes. Furthermore, Kyoto Encyclopedia of Genes and Genomes (KEGG) pathway analysis suggested a more prominent functional role for IGFBP5_tipECs than for the other EC subtypes **(**Fig. 2F**)**.

CellChat analysis **(**Fig. 2G**)** revealed augmented intercellular communication between MCs and specific EC subsets, characterized by stronger interaction intensity and an increased number of ligand-receptor pairs, with IGFBP5_tipECs exhibiting the most prominent interactions. Subsequent two-dimensional microenvironment analysis further demonstrated that in VascI^+^ LM samples, IGFBP5_ tipECs displayed markedly enhanced signal transduction activity and served as key signaling hubs, functioning as both major signal senders and receivers **(**Fig. 2H**)**. Constructing an EC cell atlas further revealed significant heterogeneity in ECs between VascI^+^ and VascI^-^ patients with CRLM.

### IGFBP5_tipECs are associated with VascI and CRLM

**Fig. 3:**
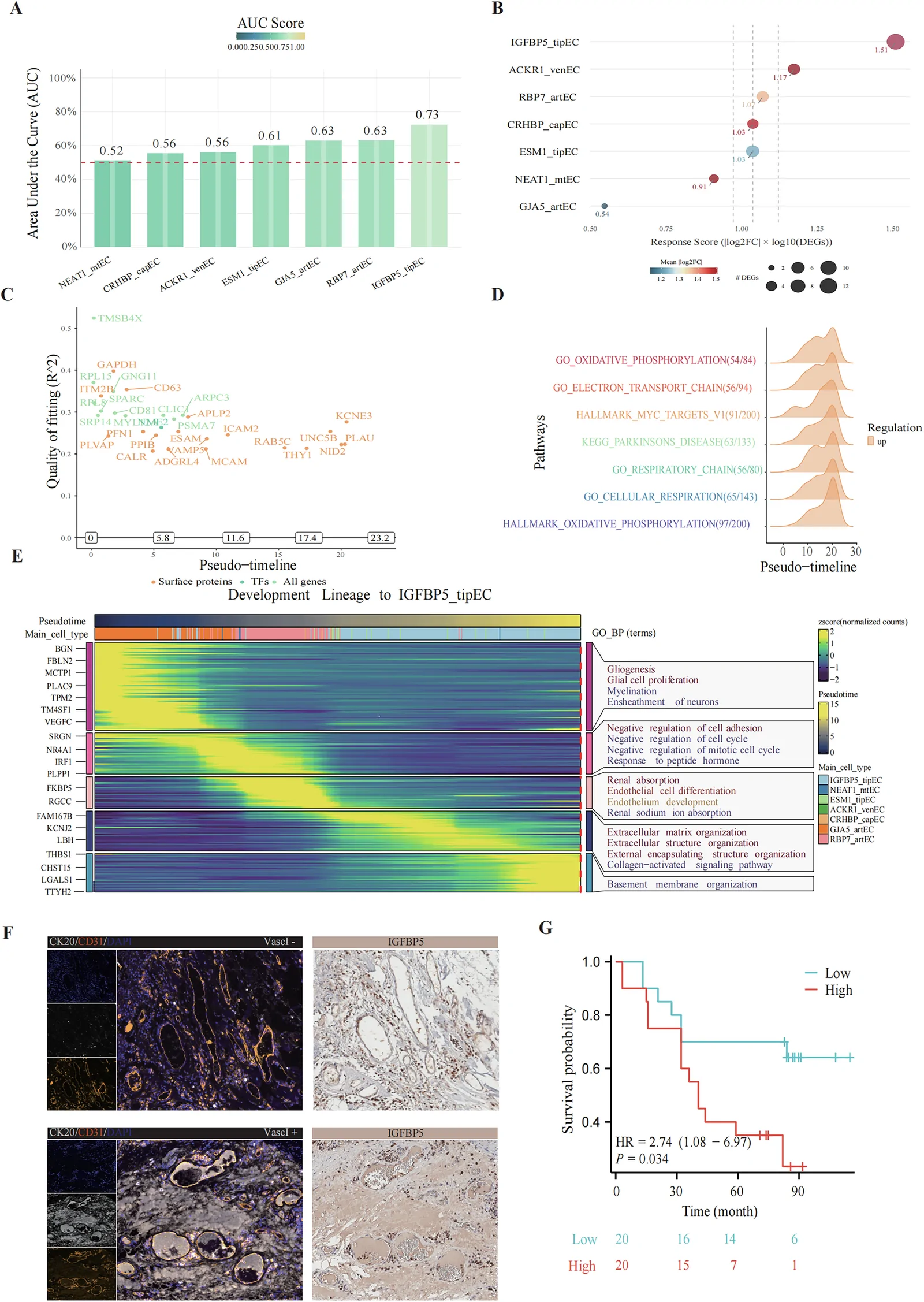
IGFBP5_tipECs are associated with VascI and correlate with poor CRC prognosis. **A** Augur algorithm analysis ranking EC subset responsiveness to VascI status, with IGFBP5_tipEC showing the strongest response. **B** Differential gene variability analysis quantifying transcriptomic reprogramming extent across EC subsets. **C** GeneSwitches analysis identifying key transcriptional regulators during the NEAT1_mtEC to IGFBP5_tipEC transition. **D** Pathway analysis revealing activated biological processes during IGFBP5_tipEC differentiation. E TradeSeq analysis of trajectory-dependent gene expression patterns along the endothelial differentiation path. **F** Representative mIF of CD31 and CK20, and IHC staining of IGFBP5 in VascI^+^ versus VascI^-^ human cancer tissues (Scale bar, 50 μm). **G** Kaplan-Meier survival analysis of CRC patients stratified by IGFBP5 expression levels. *n* = 40, Log-rank test.

Computational analysis using the Augur algorithm **(**Fig. 3A**)** and differential gene variability assessment between groups **(**Fig. 3B**)** consistently identified IGFBP5_tipECs as the most responsive to VascI status, suggesting extensive transcriptomic reprogramming within this cell population. Pseudo-three-dimensional spatial reconstruction using CSOmap **(**Supplementary Fig. 4A-D**)** demonstrated significantly enhanced interactions between IGFBP5_tipECs and MCs in VascI^+^ LM samples **(**Supplementary Fig. 4E**)** compared with those in VascI^-^ LM **(**Supplementary Fig. 4F**)**. To characterize angiogenic progression, we excluded rare outlier populations (MKI67_proECs and CCL21_lymECs) and integrated multiple trajectory algorithms (Monocle **[**Supplementary Fig. 5A**]**, Slingshot **[**Supplementary Fig. 5B**]**, and Vector **[**Supplementary Fig. 5C**]**). This analysis positioned IGFBP5_tipECs at the trajectory terminus, suggesting their activation during angiogenesis. CytoTRACE 2 analysis further validated their mature developmental status **(**Supplementary Fig. 5D-F**)**.

GeneSwitches analysis identified key transcriptional regulators involved in the transition from NEAT1_mtECs to IGFBP5_tipECs, including cytoskeletal regulators (TMSB4X, MYL12A), metabolic enzymes (GAPDH, NME2), membrane transporters (CD81, CD63), and adhesion molecules (SPARC, ICAM2) **(**Fig. 3C**)**. Pathway analysis revealed activation of oxidative phosphorylation and bioenergetic pathways, with significant involvement of electron transport chain components and cellular respiration machinery **(**Fig. 3D**)**. These findings suggest that IGFBP5^+^ tipECs differentiation involves metabolic reprogramming toward oxidative energy production, accompanied by coordinated induction of cytoskeletal remodeling and angiogenic regulators. TradeSeq analysis of trajectory-dependent gene expression **(**Fig. 3E**)** demonstrated a sequential activation pattern: initial prominence of neurodevelopmental genes, followed by upregulation of negative regulators of the cell cycle and adhesion, transitioning through endothelial differentiation and migration markers, and ultimately, extracellular matrix (ECM) remodeling and collagen adhesion pathways, consistent with the acquisition of pro-angiogenic functionality. These findings suggest that IGFBP5_tipECs contribute to VascI formation by regulating adhesion and angiogenesis in CRLM.

To validate the clinical correlation between IGFBP5 and VascI, we performed immunohistochemistry (IHC) on CRC tissue samples obtained from our patient cohort. IGFBP5 expression was found to be higher in VascI^+^ patients than in VascI^-^ patients (Fig. 3F). Survival analysis further demonstrated that higher IGFBP5 expression correlated with poorer prognosis in patients with CRC (Fig. 3G). These results indicate that IGFBP5⁺ tipECs may act as a cellular hub driving VascI formation and are associated with poorer prognosis in CRC patients.

### IGFBP5 leads to angiogenesis and barrier disruption in EC

**Fig. 4:**
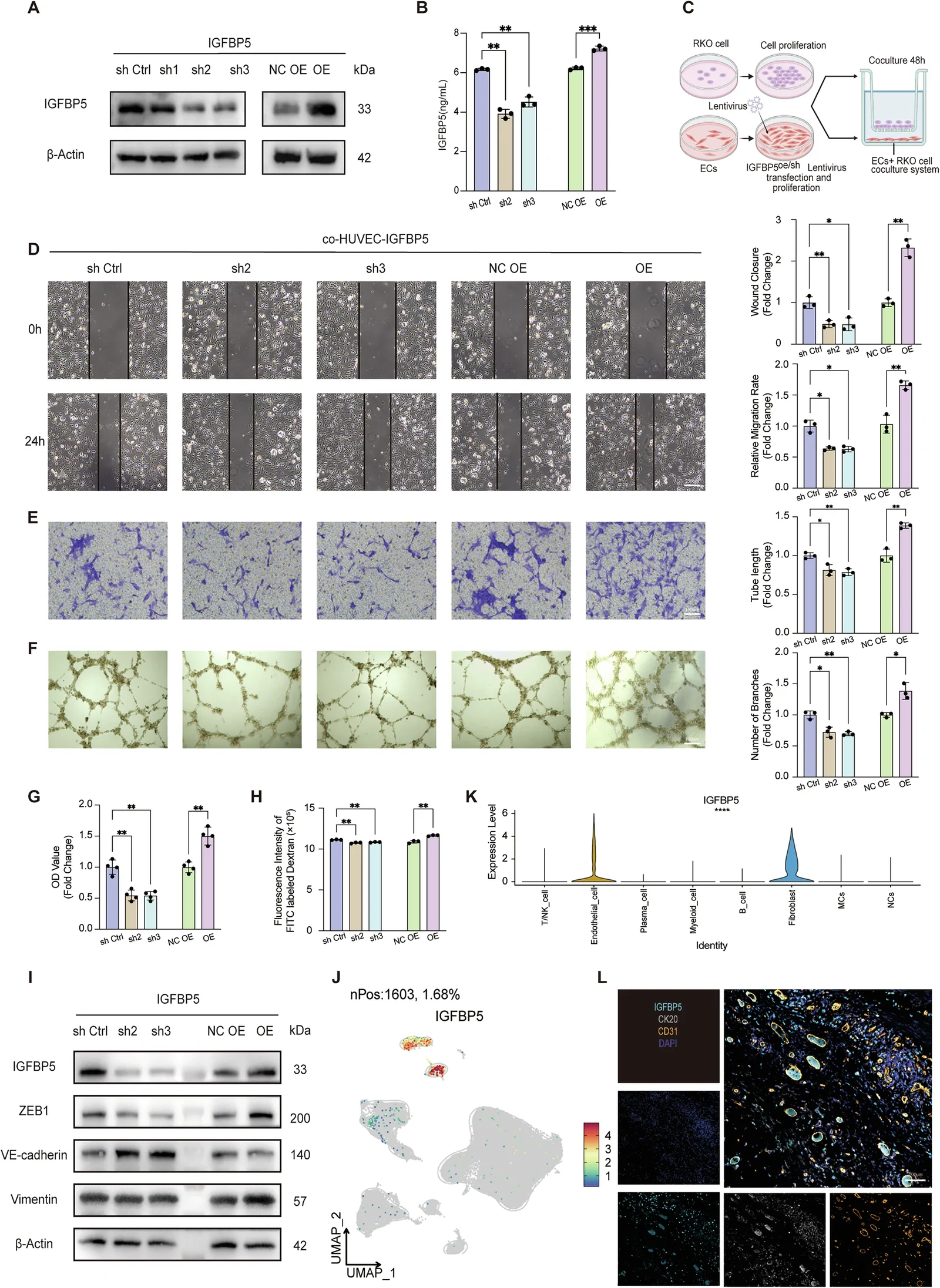
IGFBP5 leads to angiogenesis and barrier disruption in EC. **A** Western blot validation of IGFBP5 knockdown and overexpression efficiency in HUVECs. **B** ELISA quantification of secreted IGFBP5 protein levels in cell culture supernatants from IGFBP5-OE ECs and IGFBP5-sh ECs. **C** Schematic diagram of the tumor cells-ECs co-culture system used to generate tumor-associated ECs. **D**, **E** Migration capacity assessment of IGFBP5-modulated ECs by **D** wound healing assay (*n* = 3. Scale bar, 250 μm) and **E** transwell migration assay (*n* = 3. Scale bar, 100 μm). **F** Tube formation assay demonstrating angiogenic potential of IGFBP5-modulated ECs on Matrigel (*n* = 3. Scale bar,100 μm). **G** Cell adhesion assay showing ECM adhesion capability of IGFBP5-modulated ECs (*n* = 4). **H** Trans-endothelial permeability assay showing IGFBP5-OE increased the permeability of the EC monolayer (*n* = 3). **I** WB was used to detect the expression levels of endothelial marker VE-cadherin and stromal markers Vimentin and ZEB1 between different groups. **J**, **K** UMAP (**J**) and Violin plot **K** visualization of IGFBP5 expression distribution across major cell types in CRLM. **L** mIF staining showing co-localization of IGFBP5 (blue) with ECs (CD31^+^, orange) and tumor cells (CK20^+^, white) in human cancer tissues (Scale bar, 100 μm). All data are presented as mean ± SD. Statistical significance was assessed using one-way ANOVA. *P < 0.05, **P < 0.01, ***P < 0.001, ****P < 0.0001; ns not significant.

To functionally validate the role of the IGFBP5_tipEC subset identified by scRNA-seq, we generated IGFBP5-overexpressing HUVECs (IGFBP5-OE ECs) and IGFBP5 knockdown HUVECs (IGFBP5-sh ECs) as experimental models. While these engineered cells do not fully recapitulate the complexity of the endogenous tip-like subset, they allow us to assess the causal role of IGFBP5 in endothelial cell function. The transfection efficiency of the lentiviruses was verified by Western blot (WB) and quantitative real-time PCR (qRT-PCR) (Fig. 4A, Supplementary Fig. 6A). For subsequent experiments, shRNA-2 and shRNA-3 cell lines selected based on their IGFBP5 knockdown efficiency, and IGFBP5-overexpressing (IGFBP5-OE) human umbilical vein endothelial cell (HUVEC) cell lines were used. Additionally, enzyme-linked immunosorbent assay (ELISA) demonstrated that IGFBP5 secretion was significantly increased in IGFBP5-OE ECs but decreased in IGFBP5-knockdown (IGFBP5-sh) ECs (Fig. 4B). In this study, HUVECs were co-cultured with tumor cells to “educate” them into tumor-associated ECs (Fig. 4C). The optimal co-culture duration was determined by assessing IGFBP5 expression in HUVECs co-cultured with HCT116 and RKO cells. WB experiments revealed that IGFBP5 levels peaked after 48 h of co-culture with RKO cells (Supplementary Fig. 6B). Therefore, HUVECs co-cultured with RKO for 48 h were used for subsequent functional assays and RNA-seq analysis.

Results from both the wound healing and transwell assays showed that IGFBP5-OE significantly enhanced the migratory capacity of HUVECs, whereas IGFBP5-sh suppressed it (Fig. 4D, E). Matrigel tube formation assay further demonstrated that IGFBP5-OE ECs enhanced angiogenesis, while IGFBP5-sh ECs inhibited it (Fig. 4F). Additionally, adhesion assays revealed that IGFBP5 enhanced HUVECs adhesion to the ECM (Fig. 4G). The trans-endothelial permeability assay showed that IGFBP5-OE significantly increased the permeability of the EC monolayer, suggesting potential disruption of the endothelial barrier, whereas IGFBP5-sh decreased this permeability (Fig. 4H). The loss of ECs connection can lead to an increase in endothelial cell permeability. To verify this result, we detected IGFBP5 on endothelial cell marker VE-cadherin and mesenchymal markers Vimentin and ZEB1 using WB. We found that over-expression of IGFBP5 up-regulated Vimentin and ZEB1, down-regulated VE-cadherin, and vice versa (Fig. 4I). Subsequently, we performed RNA-seq analysis to elucidate the mechanism by which IGFBP5 regulates EC function. We found that 3955 genes were upregulated and 4438 genes were downregulated in the IGFBP5-OE ECs, while 498 genes were upregulated and 470 genes were downregulated in IGFBP5-sh ECs. We intersected the differentially expressed genes (DEGs) between the knockdown and OE groups and identified 579 overlapping genes (Supplementary Fig. 6C, D). Functional enrichment analysis of these DEGs was conducted. Specifically, GO enrichment analysis showed that the DEGs were predominantly associated with processes such as angiogenesis, focal adhesion, and ECM remodeling. In contrast, KEGG enrichment analysis highlighted a strong correlation with the PI3K/AKT signaling pathway (Supplementary Fig. 6E). These findings indicate that DEGs play an important regulatory role in promoting angiogenesis and activating the PI3K/AKT signaling pathway in CRC.

Furthermore, although scRNA-seq results showed that IGFBP5 was primarily expressed in ECs and CAF cells, with lower expression levels in tumor cells (Fig. 4J, K), mIF results revealed that IGFBP5 co-localized with both ECs (CD31⁺) and tumor cells (CK20⁺) (Fig. 4L). To identify the source of IGFBP5, WB was performed on ECs, CAFs, and several CRC cell lines, revealing that IGFBP5 expression was significantly lower in tumor cells and CAFs than in ECs (Supplementary Fig. 6F). In order to directly observe whether co-culture with CAF significantly affects the expression changes of IGFBP5 in tumor cells, we established a co-culture system of CAFs with HCT116 or RKO cells. And IF detection was performed on tumor cells after co-cultured for 48 h. The results showed that the expression of IGFBP5 in tumor cells was not significantly upregulated after co-cultured with CAFs. Interestingly, when CAFs were co-cultured with HUVECs, we observed a significant upregulation of IGFBP5 expression in CAFs (Supplementary Fig. 6G). These findings suggest that IGFBP5 is mainly secreted by ECs.

### IGFBP5 overexpressed ECs promote VascI formation by enhancing tumor cells proliferation, migration, and adhesion

**Fig. 5:**
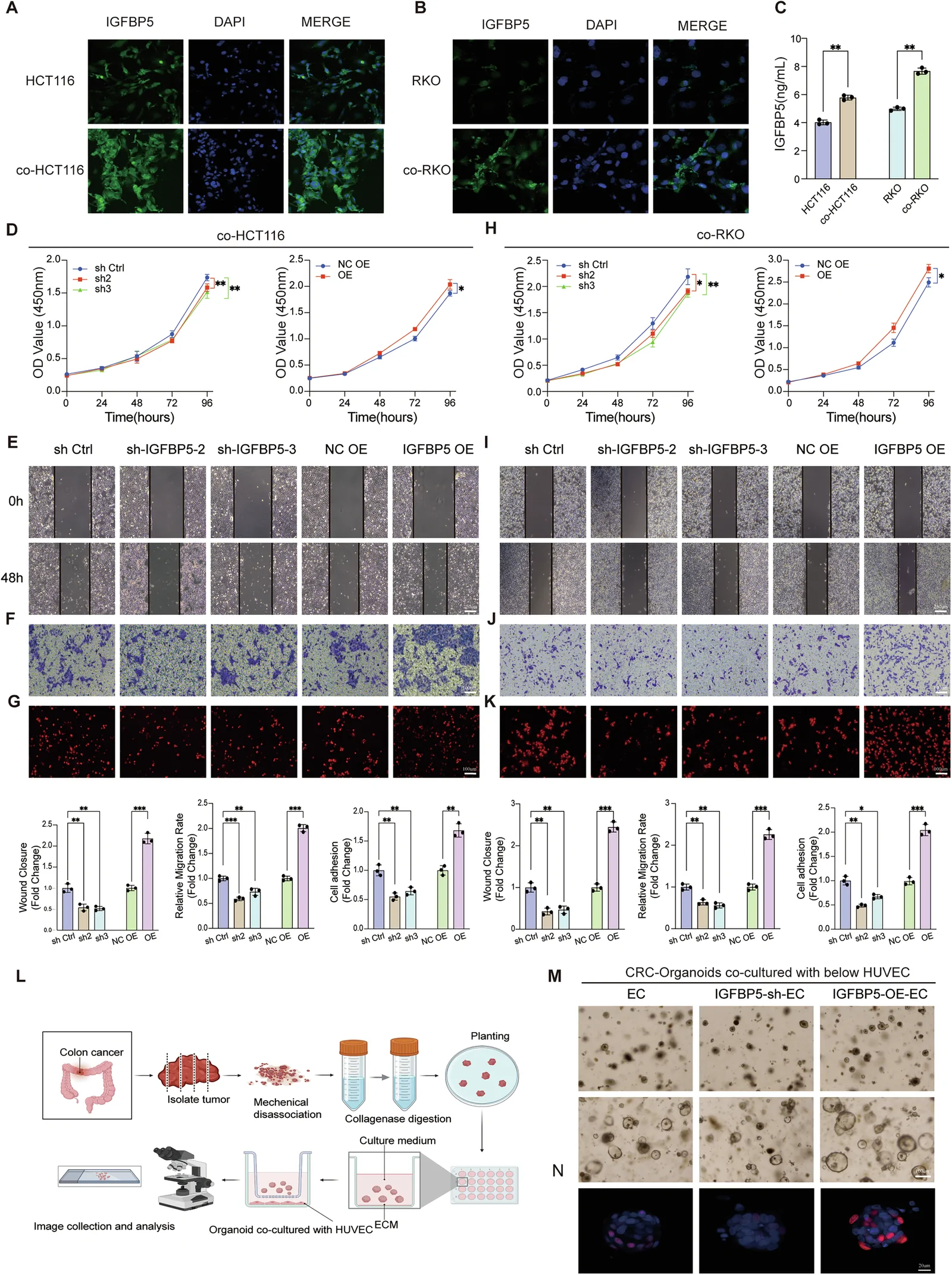
IGFBP5-OE ECs enhance tumor cells proliferation, migration, recruitment and adhesion. **A, B** Immunofluorescence staining showing IGFBP5 uptake by HCT116 **A** and RKO **B** cells after 48 h co-culture with ECs. **C** ELISA quantification of IGFBP5 levels in tumor cell supernatant after co-culture with ECs. **D**, **H** CCK-8 proliferation assays of HCT116 **D** and RKO **H** cells co-cultured with IGFBP5-modulated ECs (*n* = 5). **E, I** Wound healing migration assays of HCT116 **E** and RKO **I** cells co-cultured with IGFBP5-modulated ECs (*n* = 3. Scale bar, 250 μm). **F, J** Transwell recruitment assays of HCT116 **F** and RKO **J** cells co-cultured with IGFBP5-modulated ECs (*n* = 3. Scale bar, 100 μm). **G, K** Cell adhesion assays of HCT116 **G** and RKO **K** cells co-cultured with IGFBP5-modulated ECs (*n* = 3. Scale bar, 100 μm). **L** Flow chart of the PDO generated from CRC samples and co-cultured with HUVEC. **M** Bright-field images showing morphological changes of CRC PDOs co-cultured with IGFBP5-modulated ECs. **N** EdU incorporation assays (red) with DAPI (blue) quantifying proliferative capacity of PDOs co-cultured with IGFBP5-modulated ECs (Scale bar, 20 μm). *P < 0.05, **P < 0.01, ***P < 0.001, ****P < 0.0001; ns, not significant.

IGFBP5 is a secreted protein that can be released into the extracellular microenvironment and potentially taken up by neighboring cells. To explore changes in its expression within the co-culture system, HCT116 and RKO cells were co-cultured with ECs for 48 h. Subsequent cellular IF and ELISA analyses revealed a significant increase in IGFBP5 expression in tumor cells (Fig. 5A-C), indicating that secreted IGFBP5 can be taken up by tumor cells to exert functional effects. To assess the impact of IGFBP5-OE ECs on tumor cells, we performed CCK-8 assays on co-cultured HCT116 cells. We found that the proliferative capacity of HCT116 cells was reduced after co-culture with IGFBP5-sh ECs, whereas co-culture with IGFBP5-OE ECs produced a contrasting effect (Fig. 5D). Wound healing assays showed that co-culture with IGFBP5-OE ECs enhanced the migratory capacity of HCT116 cells, but reduced migratory capacity when co-cultured with IGFBP5-sh ECs (Fig. 5E). Similarly, transwell assays demonstrated that IGFBP5-OE ECs exerted a significant chemotactic effect on HCT116 cells and enhanced their migratory capacity, whereas IGFBP5-sh ECs suppressed them (Fig. 5F). Additionally, adhesion assays demonstrated that co-culture with IGFBP5-OE ECs markedly increased the adhesive capacity of HCT116 cells, whereas co-culture with IGFBP5-sh ECs diminished it (Fig. 5G). These results were consistent in co-cultured RKO cells (Fig. 5H-K).

Furthermore, we generated PDO from CRC samples and co-cultured with ECs in a transwell system to determine the effect of IGFBP5-OE ECs on CRC PDO, the specific process is illustrated in Fig. 5L. By observing organoid morphology and performing EdU assays, we found that IGFBP5-OE ECs significantly promoted organoid growth, whereas organoids co-cultured with IGFBP5-sh ECs exhibited relatively slower growth speed (Fig. 5M**, N**). In summary, IGFBP5 secretion by ECs enhances the proliferation, migration, and adhesion of tumor cells and recruits more tumor cells to adhere to the vascular wall, thereby promoting VascI formation.

To elucidate the mechanism by which IGFBP5-expressing ECs affect tumor cells, we performed RNA-seq on co-cultured tumor cells. Compared with the control group, 900 genes were upregulated and 1218 genes were downregulated in tumor cells co-cultured with IGFBP5-OE ECs. In contrast, 104 genes were upregulated and 198 genes were downregulated in tumor cells co-cultured with IGFBP5-sh ECs (Supplementary Fig. 6H). We then focused on DEGs which were upregulated in the IGFBP5-OE EC co-cultured group but downregulated in the IGFBP5-sh EC co-cultured group, and conduct GO and KEGG enrichment analyses. GO analysis revealed enrichments in the positive regulation of kinase activity, tumor epithelial migration, and focal adhesion, etc. KEGG pathway analysis demonstrated significant enrichment of DEGs in the PI3K/AKT signaling pathway (Supplementary Fig. 6I).

### Ligand-receptor interaction between IGFBP5 and ITGB3

**Fig. 6:**
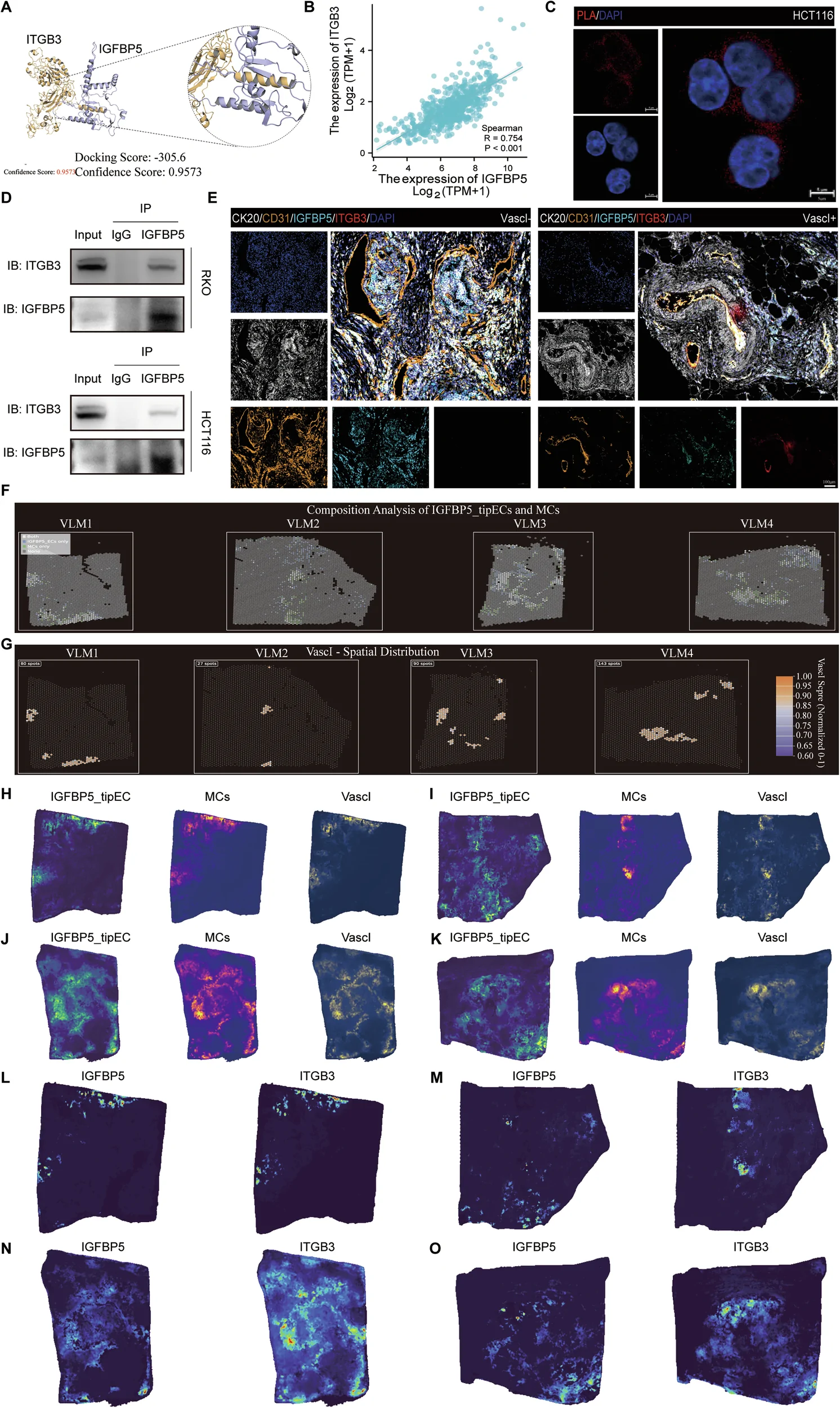
Ligand-receptor interaction between IGFBP5 and ITGB3*.* **A** Molecular docking model of IGFBP5 and ITGB3 interaction with high confidence score (0.9573). **B** Correlation analysis of IGFBP5 and ITGB3 expression in TCGA CRC cohort. **C** PLA experiment detects the interaction between IGFBP5 and ITGB3 in HCT116 cell samples (Scale bar, 5 μm). **D** Co-IP confirmed the physical interaction between ITGB3 and IGFBP5 in RKO and HCT116 cells. **E** mIF staining showing co-localization of IGFBP5 (blue) and ITGB3 (red) in VascI^+^ and VascI^⁻^ human CRC tissues (Scale bar, 100 μm). **F** ST data from four colorectal cancer liver metastasis sections (VLM1-4, dataset GSE217414) showing IGFBP5_tipEC and malignant cells (MCs). **G** ST data showing VascI spatial distribution. **H-K** Observation of co-localization between IGFBP5_tipEC, MC, and VascI using iStar algorithm. **L**, **M**, **N**, **O** Validations spatial overlap between IGFBP5 and ITGB3 at the ligand-receptor level.

To identify key genes that may mediate the interaction between IGFBP5-OE ECs and tumor cells, we intersected all DEGs from co-cultured tumor cells and ECs, identifying three overlapping genes: ITGB3, PROS1, and RF00019 **(**Supplementary Fig. 6J**)**. Among these, ITGB3 as a cell-surface receptor, may have a ligand-dependent relationship with IGFBP5. To test this, we used HDOCK molecular docking to assess the binding between ITGB3 and IGFBP5, which revealed a strong interaction affinity with a high confidence score of 0.9573 **(**Fig. 6A, Supplementary Fig. 6K**)**. Additionally, analysis of TCGA database showed a positive correlation between IGFBP5 and ITGB3 expression levels **(**Fig. 6B**)**. To explore the interaction between ITGB3 and IGFBP5 in vitro, HCT116 cells were selected and PLA experiments were conducted using anti-ITGB3 and anti-IGFBP5 antibodies. Significant PLA fluorescence signals were observed in the nucleus, indicating a direct interaction between those two proteins (Fig. 6C). Furthermore, Co-IP assays detected the specific interaction between IGFBP5 and ITGB3 (Fig. 6D). In order to better observe the co-localization phenomenon in tumor cells, we performed cellular IF after overexpression of IGFBP5 in tumor cells and found that IGFBP5 co-localized with ITGB3 both in RKO and HCT116 cells. (Supplementary Fig. 6L). In addition, to verify the in-situ binding of IGFBP5 to ITGB3, we analyzed CRC tissue sections and observed distinct co-localized fluorescence signals between IGFBP5 and ITGB3. These signals were significantly stronger in VascI^+^ CRC tissues than in VascI^⁻^ tissues (Fig. 6E).

Advances in spatial transcriptomics have enabled detailed mapping of cellular distributions within tissues. We analyzed four colorectal cancer liver metastasis sections (designated VLM1-4 in this study) from dataset GSE217414 to investigate the spatial relationship between IGFBP5_tipECs and VascI. First, using signature scoring, we characterized the spatial distributions of IGFBP5_tipECs and MCs (Fig. 6F). Spots were classified into four categories based on signature scores: negative for both signatures, IGFBP5_tipECs high only, MCs high only, or double positive. We observed that double-positive spots showed strong spatial overlap with VascI^-^ high regions (Fig. 6G). To resolve spatial patterns at higher resolution, we applied the iStar algorithm, which infers super-pixel level expression profiles by integrating histomorphological features. At near-single-cell resolution, we observed consistent and strong co-localization among the three signature scores-IGFBP5_tipECs, MCs, and VascI (Fig. 6H-K). Moreover, we validated these findings at the ligand-receptor level, demonstrating pronounced spatial overlap between IGFBP5 and its receptor ITGB3 (Fig. 6L-O). This pattern suggests that the co-localization of IGFBP5_tipECs and MCs may contribute to the process of VascI. Together, these results indicate that IGFBP5 binds to ITGB3 on tumor cells, providing insights into its role in promoting VascI and liver metastasis.

### IGFBP5 promotes VascI and CRLM by activating the ITGB3/PI3K/AKT signaling pathway

**Fig. 7:**
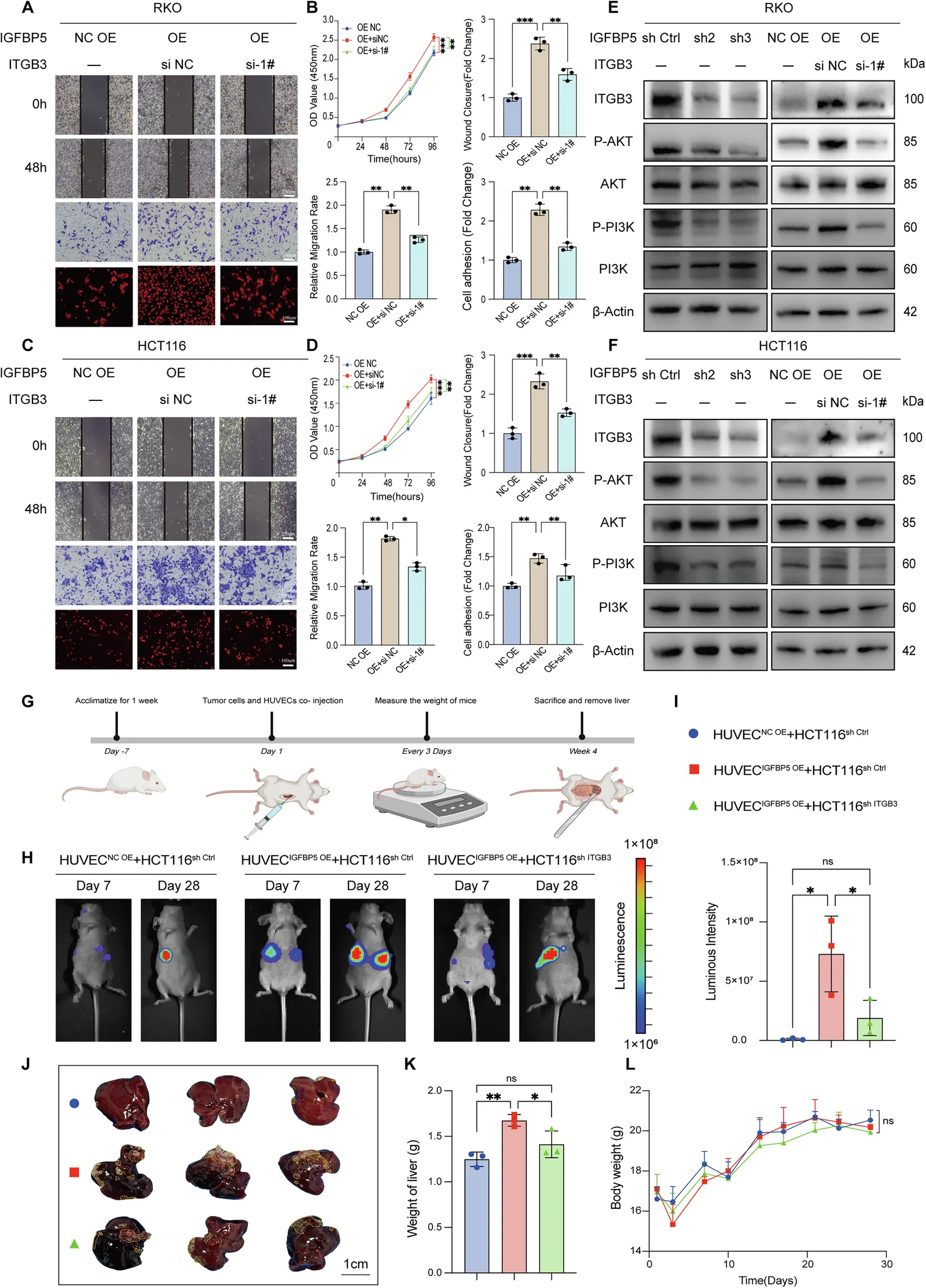
IGFBP5 promotes VascI and CRLM by activating the ITGB3/PI3K/AKT signaling pathway. **A, C** Wound healing (Scale bar, 250 μm), transwell recruitment (Scale bar, 100 μm) and cell adhesion assays (Scale bar, 100 μm) of siITGB3 RKO cells **A** and siITGB3 HCT116 cells **C** co-cultured with IGFBP5-OE ECs (*n* = 3). **B, D** CCK-8 proliferation assay of siITGB3 RKO cells **B** and siITGB3 HCT116 cells **D** co-cultured with IGFBP5-OE ECs (*n* = 5). **E, F** Western blot analysis of ITGB3/PI3K/AKT pathway components in co-cultured RKO cells **E** and HCT116 cells **F**. **G** Animal experiment flowchart and grouping information. **H, I** Representative images **H** and quantification **I** of bioluminescence signals in tumor sections (*n* = 3). **J, K** Representative images **J** and quantification **K** of liver metastases derived from CRCLM xenografts (*n* = 3). Yellow dotted lines indicate the liver metastatic foci. Scale bar, 1 cm. **L** Body weight change curves of different groups of mice. * *P* < 0.05, **P < 0.01, ***P < 0.001, ****P < 0.0001; ns not significant.

To clarify whether IGFBP5 induces phenotypic changes in tumor cells by activating ITGB3, we constructed ITGB3 negative control (ITGB3-NC) and transient ITGB3 silencing (si-ITGB3) cells via small interfering RNA (siRNA) transfection in HCT116 and RKO cells, and validated the effectiveness of ITGB3 transient knockdown using WB and qRT-PCR (Supplementary Fig. 6M, N). We found that in ECs co-culture system, compared with ITGB3-NC tumor cells, silencing ITGB3 resulted in reduced proliferation, migration, recruitment, and adhesion abilities in HCT116 and RKO (Fig. 7A-D). These results suggest that IGFBP5 may exert its effects through ITGB3 activation.

To further elucidate the molecular mechanism by which IGFBP5-OE ECs promote CRC cell progression, KEGG and Gene Set Enrichment Analysis (GSEA) enrichment analyses were performed based on the RNA-seq results. Both analyses revealed positive regulation of focal adhesion and the PI3K/AKT signaling pathway (Supplementary Fig. 6O). This finding suggests that the PI3K/AKT signaling pathway plays a critical role in VascI formation. Therefore, we assessed the protein expression levels of PI3K, phosphorylated-PI3K (p-PI3K), AKT, and phosphorylated-AKT (p-AKT) in co-cultured tumor cells to validate the regulatory effect of IGFBP5 expressing ECs on the ITGB3/PI3K/AKT signaling pathway. Co-cultured with IGFBP5-sh ECs inhibited ITGB3 expression and the phosphorylation of PI3K and AKT, whereas co-cultured with IGFBP5-OE ECs activated this pathway. However, ITGB3-silenced tumor cells partially inhibited PI3K/AKT pathway activation in co-cultured system (Fig. 7E, F). These results suggest that IGFBP5-OE ECs promote VascI formation and CRLM through the ITGB3/PI3K/AKT signaling pathway.

To determine whether IGFBP5 derived from ECs exerts its pro metastatic effect in vivo through ITGB3, we established a CRLM model by co-injection of ECs and tumor cells in BALB/C nude mice. The specific grouping and experimental process are shown in Fig. 7G. Remarkably, receiving HUVEC ^IGFBP5 OE^+HCT116^sh Ctrl^ group notably facilitated liver metastasis compared to the HUVEC^NC OE^+HCT116^sh Ctrl^ group. However, after knocking down the ITGB3 on HCT116, the pro-metastasis role of HUVEC^IGFBP5 OE^ was significantly reduced (Fig. 7H-K**, L**). Collectively, these in vivo findings align closely with our functional studies, demonstrating that IGFBP5 derived from ECs drive liver metastasis through activation of ITGB3 on tumor cells.

## Discussion

LM, the leading cause of CRC-related mortality, is a highly selective process in which only a small subset of tumor cells can survive the hostile vascular environment and successfully extravasate to form secondary tumors [24]. VascI significantly enhances the efficiency of this process and is considered a key determinant of adverse clinical outcomes [14]. In this study, we demonstrated that VascI is an important prognostic factor for CRLM by analyzing 40 patients with CRC from our hospital and 229 patients from TCGA database. Despite receiving curative treatment, patients with CRC and VascI exhibit a higher risk of developing CRLM and a poorer prognosis. However, the precise mechanisms underlying tumor cell adhesion and infiltration within blood vessels remain unclear. Therefore, through scRNA-seq analysis and in vitro experiments, this study identified a novel molecular axis that mediates the crosstalk between ECs and tumor cells to drive this process. Specifically, IGFBP5 (secreted by tipECs), binds to integrin ITGB3 on the surface of both tumor cells and ECs via paracrine and autocrine secretion, activates the PI3K/AKT signaling pathway, and promotes VascI and CRLM. These findings provide mechanistic insights into the development of VascI and highlight potential therapeutic targets for inhibiting metastasis.

ECs possess tube-forming capacity and play a crucial role in angiogenesis, a process closely correlated with cancer progression and metastasis [25]. In our study, a tipEC subcluster (High expression of IGFBP5) was significantly enriched in CRLM with VascI, indicating the pro-metastatic potential of IGFBP5_tipECs. IGFBP5 is a secreted protein and a member of the insulin-like growth factor (IGF)-binding protein family, a group of proteins capable of binding IGF and exerting two-sided effects on IGF-1 and IGF-2 [26]. Among these proteins, IGFBP5 is the most highly conserved member [27, 28]. Although IGFBP5 mainly regulates IGF activity, it also has multiple IGF-independent biological functions, including the regulation of inflammatory response, cell adhesion, cell migration, cell proliferation, fibrosis, epithelial cell development, cell senescence and ECM remodeling, and EndMT [29–31]. The upregulation of IGFBP5 expression has been reported in both UC and CRC tissues [32]. Our findings demonstrate that IGFBP5 overexpression involves profound transcriptional reprogramming of ECs, characterized by progressive loss of canonical endothelial features (angiogenesis, cell-cell junction maintenance and classic inflammatory adhesion pathway) and concurrent acquisition of mesenchymal effector functions (such as migration, ECM remodeling, collagen deposition, and integrin activation). This phenotypic conversion recapitulates core features of EndMT. These findings suggest that IGFBP5 may mediate cancer associated HUVECs migratory capacity, markedly increased angiogenic abilities while disrupting vessel integrity. Although we also found that CAF provides IGFBP5, it may play a secondary or context dependent role in the TME. However, our results indicate that EC is the main source of IGFBP5. We also highlight its key functions in VascI and pro-metastatic processes. Collectively, this study characterizes the TME of VascI in CRLM and investigates how IGFBP5 derived from ECs drive VascI formation, providing a potential target for CRLM management.

Beyond its classic role of indirectly regulating IGF1R activity via competitive binding to IGF-1 or IGF-2, IGFBP5 also interacts with receptors such as ROR1 (receptor tyrosine kinase-like orphan receptor 1) and RARα (retinoic acid receptor alpha) [33, 34]. However, the interaction between IGFBP5 and ITGB3 is an unknown mechanism of intercellular crosstalk. In this study, we identified ITGB3 as a gene that is significantly regulated by IGFBP5 through RNA-seq and predicted its potential binding ability using molecular docking. Although direct binding between IGFBP5 and ITGB3 has not been experimentally validated, our results provide strong indirect evidence consistent with previous studies. Nguyen et al. identified DEGs between homozygous transgenic mice and wild-type mice using RNA-seq and found that 7% were receptors and integrins [28]. Berfield et al. found that IGFBP5-mediated mesangial cell migration was inhibited by antibodies that block attachment to α6β1-integrins [35]. Consistently, we observed through WB, mIF, and spatial transcriptome data and revealed that IGFBP5 may exert its function by directly binding to ITGB3, and ITGB3 is essential for tumor cell attachment to vessels, thereby enhancing VascI and promoting cancer metastasis. These results provide in-depth mechanistic insights into the crosstalk between tumor cells and ECs in driving angiogenesis and the disruption of endothelial barrier function, which is crucial for designing more targeted and effective therapies against tumor VascI and CRLM.

RNA-seq and bioinformatics analyses revealed that the PI3K/AKT signaling pathway was the most significantly enriched in both IGFBP5-OE ECs and tumor cells co-cultured with IGFBP5-OE ECs. This finding suggests that the PI3K/AKT signaling pathway may serve as a downstream mediator of the IGFBP5-ITGB3 axis in both tumor cells and ECs, playing an important role in CRLM. The PI3K/AKT signaling pathway is a well-established central regulator of tumorigenesis and metastasis, promoting cell survival, proliferation, and metabolic adaptation under stressful conditions [36, 37]. In angiogenesis, PI3K/AKT activation induces vascular endothelial growth factor (VEGF) production in cancer cells [38]. VEGF binding to its receptor activates PI3K signaling in ECs, triggering actin reorganization and cell migration, thereby enhancing angiogenesis [25]. Hyperactivation of this pathway is frequently observed in CRLM and is strongly associated with accelerated metastasis, enabling CTCs to survive in harsh vascular microenvironments and colonize secondary sites [39]. IGFBP5 expression is upregulated in patients with CRC and correlates with tumor differentiation, lymph node metastasis, drug resistance, prognosis, and involvement in the PI3K/AKT signaling pathway [40]. Tong et al. found that IGFBP5 promotes AKT1 phosphorylation (pT308AKT1/total AKT1). Additionally, Yang et al. reported that HOXD3 overexpression activates MAPK/ERK and AKT signaling by upregulating integrin β3 expression, thereby enhancing CRC growth and metastasis [41]. These studies provide indirect evidence of crosstalk between IGFBP5, ITGB3, and the PI3K/AKT signaling pathway.

Several limitations of this study should be acknowledged. First, our study conducted functional experiments by co-culturing HUVECs with tumor cells to “educated” them into tumor associated endothelial cells. Ideally, isolating primary IGFBP5_tipEC would be better. However, IGFBP5 is a secreted protein, which makes it technically unfeasible. To partially address this gap, we performed mIF on human CRC tissues, confirming the presence and spatial distribution of IGFBP5 in the actual tumor microenvironment. Second, the crosstalk between the PI3K/AKT pathway and other potential downstream effectors, such as MAPK and mTOR, requires further investigation. Third, investigating the universality of the IGFBP5/ITGB3/PI3K/AKT axis in other cancer types with high VascI potential, such as gastric cancer or hepatocellular carcinoma, would be valuable to expand the translational relevance of our findings.

### Conclusion

In summary, we identified a novel EC-driven IGFBP5/ITGB3/PI3K/AKT signaling axis that is pivotal for VascI formation in CRC. This study advances our understanding of the dynamic crosstalk between tumor cells and their vascular niche. It provides a conceptual framework for therapeutic strategies aimed at suppressing metastasis by targeting the crucial steps of tumor VascI. Therefore, greater attention should be directed toward investigating the biology and molecular mechanisms of VascI in CRC to develop timely, effective, and comprehensive therapeutic strategies for patients with CRC and VascI.

## Methods

### Patients and follow-up

We collected the clinical data on VascI from two cohorts of patients with CRC: 229 cases from TCGA with documented VascI status, and 40 pathologically diagnosed patients with CRC (26 males and 14 females) treated at the Cancer Hospital, Chinese Academy of Medical Sciences and Peking Union Medical College between January 1, 2015, and December 12, 2019. Detailed clinical characteristics and follow-up information of these patients are provided in Table S1. All patient specimens collected in this study were obtained from an East Asian population. This study was approved by the Ethics Committee of the Cancer Hospital, Chinese Academy of Medical Sciences (ID: 21/197-2868).

### Evaluated the VascI status

We used HE and mIF staining to evaluate tumor tissues, which were assessed by two experienced pathologists. The presence of cancer cell nests within blood vessels under a microscopic examination was defined as VascI, identified by the co-localization of CD31 and CK20 in mIF. Based on the Invasion status, patients were classified into two groups: VascI^+^ and VascI^-^.

### Sample acquisition, scRNA-seq workflow, and analysis

scRNA-seq was performed on surgically resected LM specimens obtained from eight treatment-naïve patients with CRLM. Patient demographics are detailed in Supplementary Table S2. Samples were categorized based on VascI status into VascI^+^ LM (*n* = 4) and VascI^-^ LM (*n* = 4) cohorts.

Low-quality cells were excluded if they expressed fewer than 3 genes, had fewer than 50 total transcripts, or exhibited high mitochondrial (≥20%) or hemoglobin (≥5%) gene content. The data were normalized and variance-stabilized using SCTransform, followed by batch-effect correction with Harmony and dimensionality reduction using PCA [42, 43]. Cell clusters were identified using shared nearest-neighbor graph-based community detection and visualized using UMAP. Marker genes for each cluster were identified to annotate cell types, and iterative sub-clustering was applied to delineate transcriptionally distinct subpopulations. Comprehensive details regarding sample processing are provided in the Supplementary Methods.

### Comparing cell type proportions between groups

To assess the differences in cellular composition within the TME between groups, we employed the Ro/e metric to quantify the enrichment (Ro/e > 1) or depletion (Ro/e < 1) of individual cell clusters [44]. The results were further validated using MiloR, a specialized method for detecting differential abundances of cell populations [45]. Subsequently, cell type proportions for distinct subsets were directly compared between the groups, with statistical significance determined using standard thresholds. Additionally, we implemented Augur, a machine learning framework that ranks cell types by quantifying transcriptional response magnitudes to biological perturbations, thereby identifying the most responsive cellular populations under defined experimental conditions [46].

### Chromosomal CNV estimation

Chromosomal CNVs were detected using the InferCNV computational tool [47]. To distinguish malignant cells from normal epithelial cells, CNV scores were calculated by integrating copy number profiles across all cellular subclusters.

### Functional enrichment analysis

To characterize the functional heterogeneity of distinct cellular subpopulations, we performed gene set enrichment analysis using irGSEA to evaluate established transcriptional signatures for epithelial cells, as previously defined [48, 49]. DEGs were screened using Seurat’s FindMarkers function and Wilcoxon rank-sum tests with default parameters. Subsequent GSEA was used to determine activated or suppressed pathways based on GO and KEGG reference sets from the MSigDB repository (https://www.gsea-msigdb.org).

### Pseudospace construction

To determine the spatial relationships among cell subpopulations in the scRNA-seq data, the CSOmap algorithm was applied [50]. This method estimates cellular proximity by analyzing ligand-receptor pair interactions and embeds cells into a 3D spatial framework.

### Cellchat analysis

Intercellular communication among distinct cell clusters within the TME was investigated using the CellChat package [51]. CellChat assesses such communication by leveraging a biologically accurate ligand-receptor-cofactor database. It quantitatively reconstructs signaling networks from scRNA-seq data, classifies pathways via manifold learning, identifies conserved or context-specific signals, and predicts cellular inputs and outputs through network analysis.

### Cell trajectory inference

Cell trajectory analysis was conducted using Slingshot, Monocle, and Vector algorithms [52–54]. Additionally, Geneswitches was implemented on pseudotime trajectories of target cell clusters to delineate critical transitions in gene expression dynamics and pathway enrichment during developmental progression [55]. CytoTRACE was subsequently applied to deduce the cellular differentiation hierarchies [56].

### IHC and IF

IHC: Formalin-fixed, paraffin-embedded human cancer tissue sections were dewaxed in xylene, rehydrated through a graded ethanol series, and subjected to antigen retrieval in EDTA buffer (pH 8.0). After blocking for 1 h at room temperature, sections were incubated with primary antibody overnight at 4 °C. The following day, sections were equilibrated to room temperature for 15 min and incubated with a horseradish peroxidase-conjugated secondary antibody for 60 min at room temperature. Finally, sections were developed using DAB, counterstained with hematoxylin, and examined microscopically.

Tissue IF: Sections underwent antigen retrieval as described for IHC, permeabilized with 0.3% Triton X-100 for 15 min at room temperature, and then blocked and incubated with primary antibodies as in IHC. Sections were incubated with fluorescent-conjugated secondary antibodies for 60 min at room temperature in the dark, counterstained with DAPI for 5 min (dark), mounted with anti-fluorescence quenching medium, and observed under a Zeiss laser scanning confocal microscope.

Cellular IF: Pretreated cells were fixed with 4% paraformaldehyde and blocked for 1 h at room temperature, followed by incubation with primary antibodies overnight at 4 °C. Cells were then incubated with Alexa Fluor-conjugated secondary antibodies for 1 h at room temperature, stained with DAPI, and examined using a Zeiss laser scanning confocal microscope.

All steps were followed by three PBS washes. Primary antibodies include, IGFBP5 polyclonal antibody (1:800, 55205-1-AP, RRID: AB_2736835, Proteintech, China), ITGB3 monoclonal antibody (1:2500, 66952-1-Ig, AB_2128759, RRID: AB_2128759, Proteintech, China), CD31 (1:3000, 11265-1-AP, RRID: AB_2299349, Proteintech, China), CK 20 Polyclonal antibody (1:2000, 17329-1-AP, RRID: AB_2133592, Proteintech, China).

### Cell line and cell culture

All cell lines in our study were obtained from Shanghai YuBio Biotech (Shanghai, China). HUVECs were purchased from YuBio Biotech (Shanghai, China), and cultured in ECM medium (ScienCell, USA) supplemented with 5% fetal bovine serum (FBS; Gibco, USA), 1% growth factor, and 1% penicillin/streptomycin (P/S; Gibco, USA), within 10 generations used for experimentation. HCT116 (purchased from Starfish Biotechnology, Suzhou, China) and RKO (purchased from YuBio Biotech, Shanghai, China) cells were cultured in Iscove’s Modified Dulbecco’s Medium (IMDM; BasalMedia, China) supplemented with 10% FBS and 1% P/S. All cell lines were authenticated by short tandem repeat (STR) profiling and tested negative for mycoplasma contamination before use. The remaining cells used to detect IGFBP5 expression in this study were sourced from our laboratory’s previously established inventory and were routinely confirmed to be free of mycoplasma contamination prior to experimental use. Cells were cultured at 37 °C in a humidified 5% CO_2_ incubator.

### siRNA transfection and lentiviral transduction

Briefly, 5 × 10^5^ cells were seeded into 6-well plates and cultured overnight to approximately 70% confluence. IGFBP5 and ITGB3 lentiviral vectors were packaged according to the manufacturer’s instructions (GeneChem, Shanghai, China), and the sequences are provided in Table S3. siRNA oligonucleotides were designed and synthesized by GenePharma (Suzhou, China). Transient transfections were performed using the Lipofectamine 3000 kit (Invitrogen, USA), following the manufacturer’s instructions. The siRNA sequences are listed in Table S4. Transfection efficiency was evaluated 48–72 h post-transfection using qRT-PCR and WB, as described in the Supplementary Methods.

### Cell co-culture system

Approximately 1 × 10^5^ tumor cells per well were seeded into the upper compartment, and 5 × 10^4^ HUVECs per well were seeded into the lower chamber of 0.4 μM transwell inserts in 6-well plates (Corning, USA). After 48 h of co-culture, both tumor cells and HUVECs were harvested for subsequent experiments.

### ELISA

The secretion levels of IGFBP5 in different HUVEC groups were determined using ELISA. HUVECs were cultured to approximately 70% confluence in ECM, washed with PBS, and cultured in FBS-free ECM for 24 h. Subsequently, the culture medium was collected and analyzed for IGFBP5 detection using a commercial ELISA Kit (RayBiotech, US).

### CCK-8

Tumor cells were seeded into 96-well plates at a density of 2 × 10^4^ /mL to 5 × 10^4^ /mL. Upon reaching the appropriate confluence, the cells were treated according to the experimental groups. At designated time points, 10 μL of CCK-8 solution (Beyotime, China) was added to each well and incubated for 2 h at 37 °C in a 5% CO_2_ incubator. Absorbance was measured at 450 nm using a phase-contrast microplate reader. Each group included five replicate wells and one blank control.

### Wound healing and transwell assays

For wound healing assays, approximately 1 × 10⁶ cells were seeded in 6-well plates (Corning, USA). The cells were incubated for 24 h and then scratched with sterile 10 μL pipette tips to create a wound. Each well was replaced with 1 mL conditioned medium. Cells were photographed at 0 and 48 h using a microscope. Residual wound area was quantified using ImageJ in ≥3 randomly selected fields. For HUVEC transwell assays, approximately 5 × 10⁴ cells suspended in FBS-free ECM were seeded into the upper chambers of 24-well plates (Corning, USA), with 800 μL of conditioned medium added to the lower chambers. For tumor cell transwell assays, approximately 1 × 10⁵ tumor cells suspended in FBS-free IMDM were seeded into the upper chambers of 24-well plates. HUVECs were seeded into the lower compartments of the same 24-well plates and supplemented with 800 μL of ECM. After 24–48 h, the membranes were fixed with 4% paraformaldehyde, stained with 0.1% crystal violet solution, and counted in ≥3 random fields with ImageJ (*n* = 3).

### Adhesion assays

Cell-ECM adhesion assay: 96-well plates coated with 10 µg/mL fibronectin (MedChemExpress [MCE], China) at room temperature for 1 h. Then, 200 μL of heat-denatured 1% bovine serum albumin (BSA) was added and incubated at 37 °C for 1 h. The plates were washed twice with FBS-free medium. A total of 5 × 10⁴ HUVECs were seeded into the pretreated 96-well plates. The cells were cultured in the incubator for 4 h, and non-adherent cells were washed away with PBS three times. The number of cells in the 96-well plates was detected using the CCK-8 method (*n* = 4).

Cell-cell adhesion assay: HUVECs (5 × 10⁴ per well) were seeded into 24-well plates and cultured to approximately 100% confluence. Subsequently, tumor cells stained with Dil red fluorescent dye (Beyotime, China) were resuspended in 500 μL of conditioned ECM and added to the pre-cultured HUVEC monolayer for 4 h of incubation. The plates were then gently washed with PBS to remove non-adherent cells. The adhesion of tumor cells to HUVECs was observed using a fluorescence microscope. Counted in ≥3 random fields with ImageJ (*n* = 3).

### Tube formation assays

Reduced growth factor Matrigel (Corning, USA) was mixed with FBS-free ECM at a 1:1 ratio. Subsequently, 50 μL of the mixture was added to each well of a 96-well plate and incubated overnight at 37 °C. HUVECs (2 × 10⁴) resuspended in 100 μL of FBS-free ECM were then seeded onto the Matrigel-coated wells. Vascular-like structures were imaged under an inverted microscope at 6 h. Tubelike structures were imaged using an inverted microscope, and total tube length and number of branches was quantified with ImageJ (*n* = 3).

### Endothelial permeability assays

EC permeability was determined using fluorescein isothiocyanate (FITC)-dextran (MW 4000; Beyotime, China), as previously described [57]. HUVECs were inoculated into transwell chambers and incubated for 72 h until confluence. After a dense EC monolayer was formed, the culture medium was replaced with fresh ECM containing FITC-dextran, while 600 µL of ECM was added to the lower chambers. After 1 h, 100 μL of culture medium was collected from the lower chambers and transferred to a black-bottom 96-well plate; an equal volume (100 μL) of fresh medium was then added back to the lower chambers to maintain volume consistency. Fluorescence intensity was measured using a microplate reader at appropriate excitation and emission wavelengths (*n* = 3).

### Construction of PDO, co-culture with HUVEC, and EdU staining

Human CRC tissues were obtained from patients undergoing curative surgical resection at our hospital. Details of the PDO construction process are provided in the Supplementary Methods. Cells and crypt fragments suspended in Matrigel (BioGenous, China) were inoculated into 24-well plates and cultured in a customized human colon organoid medium (BioGenous, China) at 37 °C and 5% CO_2_. Organoid formation was monitored daily, and the culture medium was replaced with fresh medium on alternate days.

Organoids were harvested as described in the Supplementary Methods, resuspended in Matrigel, and seeded into the upper chambers of 24-well plates. HUVECs were seeded into the lower chambers, and a 1:1 mixture of organoid culture medium and ECM was added to establish an organoid-HUVEC co-culture system. For 5-ethynyl-2’-deoxyuridine (EdU) staining, organoids were incubated with 0.05% trypsin-EDTA on ice until Matrigel was completely removed, while minimizing disruption of organoid integrity. The dissociated cells were then incubated with 2× EdU working solution (Beyotime, China). After three washes with PBS, a click reaction mixture was added to label EdU-incorporated DNA, and nuclei were counterstained with Hoechst 33342 [58]. Images were captured using a Zeiss LSM 880 confocal microscope to assess proliferative activity.

### Bulk-RNA sequencing and data analysis

Total RNA was extracted from co-cultured HUVECs and RKO cells using the TRIzol reagent and subjected to mRNA library construction. Libraries were sequenced using an Illumina platform (GeneChem, Shanghai, China). Following sequencing, RNA-seq data were aligned to the human genome, and transcripts were assembled from the alignments. Normalization and DEG analyses were performed using the DESeq2 R package [59]. Based on the identified DEGs, further GO enrichment analysis, KEGG pathway analysis, and GSEA were conducted to identify potential mechanisms.

### Molecular docking

The interaction between IGFBP5 and ITGB3 was predicted through molecular docking using HDOCKlite v1.1 tool to predict the binding affinities of protein to protein. The protein structures of IGFBP5 and ITGB3 was obtained from UniProt, and the full-length AlphaFold predicted structure of IBP5-HUMAN (Uniprot ID: P24593) was selected as the ligand protein, while the EM structure 8GCD of ITB3-HUMAN (Uniprot ID: P05106) was selected as the receptor protein. HDOCK uses a scoring function to rank the sampling process and binding patterns after sampling for proteins and protein structures.

### PLA

Anti-IGFBP5 and anti-ITGB3 antibodies were used to generate PLA probes using Duolink InSitu Probemaker kit (Sigma-Aldrich). µ-Slide Angiogenesis (Ibidi) were used to seed HCT116 cells (1×105 cells) and incubated overnight in a cell culture incubator. Then, the cells were fixed with 4% paraformaldehyde solution in PBS for 20 min at room temperature, wash two times with PBS, permeabilized with 0.5% saponin (Sigma-Aldrich) in PBS for 30 min (15 min at 4 °C and 15 min at room temperature) and blocked with Duolink blocking solution (Sigma-Aldrich) for 1 h at 37 °C. Dilute primary antibodies (anti-IGFBP5 and anti-ITGB3) in Duolink R antibody dilution buffer and stain overnight at 4 °C. PLA was performed according to the manufacturer’s instructions (Sigma-Aldrich). Then mount slides with DAPI resistant to fluorescence quenching, and examined using a Zeiss laser scanning confocal microscope.

### Co-immunoprecipitation (Co-IP)

Cells were lysed in NP-40 lysis buffer (Beyotime). Lysates were centrifuged at 12,000×g for 15 min at 4 °C, and the supernatants were collected. For each IP reaction, 500 μL of total lysate (containing approximately 1 mg of total protein) was incubated with 4 μg of anti-IGFBP5 antibody or normal rabbit IgG (as negative control) overnight at 4 °C under gentle rotation. Protein A/G agarose beads (30 μL) were then added and incubated for another 4 h. The beads were washed three times with ice-cold lysis buffer. Bound proteins were eluted by boiling in 40 μL 1×SDS loading buffer. For the Input control, 20 μL of the same lysate (representing 4% of the total lysate used for each IP reaction) was taken and mixed with SDS loading buffer. All samples (Input and IP eluates) were separated by SDS-PAGE, transferred to PVDF membranes, and immunoblotted with antibody. The ECL substrate was evenly applied to the membrane, and chemiluminescent signals were captured with identical exposure times for all blots within each independent experiment. Three independent biological replicates were performed, and a representative blot is shown. Original data for the Western blots are reported as WB Original Data.

### Spatial transcriptomics analysis of IGFBP5_tipECs

Four 10x Genomics Visium spatial transcriptomics samples of colorectal cancer liver metastases (CRLMs) were obtained from the public dataset GSE217414. Raw data were processed using Scanpy (v1.9.0). Three gene signatures were defined: an IGFBP5_tipEC signature (top 30 differentially expressed genes identified in this study), a malignant cell (MC) signature, and a vascular invasion (VascI) signature (**Supplementary Methods**). Signature scores were calculated for each spot based on z-normalized expression of detected signature genes and smoothed over spatial neighbors to incorporate local tissue context.

To identify high-confidence signature-positive regions, we applied a double-threshold filter requiring both spot-intrinsic score > 0.7 and mean score of the six nearest neighbors > 0.7. Colocalization between IGFBP5_tipEC and MC hotspots was quantified by overlapping regions meeting this threshold. For super-resolution tissue architecture inference, the iStar algorithm was employed to deconvolve spot-level data into near single-cell resolution, enabling fine-mapping of signature distribution and ligand-receptor colocalization patterns [60].

### In vivo experiments

BALB/c nude male mice (4 weeks old) were purchased from SPF Biotechnology Co., Ltd (Beijing, China). Before tumor cells injection, all mice were acclimated to specific pathogen-free (SPF) conditions for one week prior to the experiment. HCT116 cells were engineered to stably express firefly luciferase for in vivo tracking. HCT116 cells (1 × 10^6^) and HUVECs (1 × 10^6^) were suspended in 50 µL PBS and then co-injected into the spleens of mice under anesthesia. The experimental groups were as follows: HUVEC^NC OE^ + HCT116^sh Ctrl^, HUVEC^IGFBP5 OE^ + HCT116^sh Ctrl^, and HUVEC^IGFBP5 OE^ + HCT116^sh ITGB3^. Tumor progression was monitored using an in vivo imaging system. D-fluorescein potassium salt (150 mg/kg) was administered via intraperitoneal injection 10 min prior to imaging. Mouse bioluminescence was detected and quantified using Living Image software. Mice were monitored for 28 days after injection. At the end of the observation period, mice were euthanized, and livers were harvested, weighed, and photographed. This study was approved by the Institutional Animal Care and Use Committee (IACUC) of Beijing Life Bioscience Co., Ltd. (approval number: BJLF-DWLL-2025-07-02-01).

### Statistical analysis

All statistical analyses were performed using GraphPad Prism, SPSS, or Xiantao software. Data are presented as mean ± SD from at least three independent experiments. Statistical significance was determined using a two-tailed Student’s t-test, one-way ANOVA, two-way ANOVA or Mann–Whitney U test, as appropriate, and set at *P* < 0.05. Survival analysis was conducted using Kaplan-Meier curves and the log-rank test.

## Supplementary information

Supplementary Methods

Supplementary Figures

Supplementary Tables

Original WB

## Data Availability

The RNA-seq data supporting the results of this study have been deposited in the Gene Expression Omnibus (GEO) under accession number (GSE333995). The spatial transcriptomics data (GSE217414) are publicly available from GEO. The TCGA data used in this study are accessible via the GDC data portal (https://portal.gdc.cancer.gov/). Other datasets or additional information are available from the corresponding author upon reasonable request.
