# Supplementary material for: IGFBP5 derived from endothelial cells drive vascular invasion and liver metastasis of colorectal cancer by activating the ITGB3/PI3K/AKT Axis
Source: Cell Death Dis. 2026 Jul 23;17(1):891. doi: 10.1038/s41419-026-09081-6 (PMC13680512; doi:10.1038/s41419-026-09081-6)
Supplement: Supplementary file 1 [file 41419_2026_9081_MOESM4_ESM.pdf]

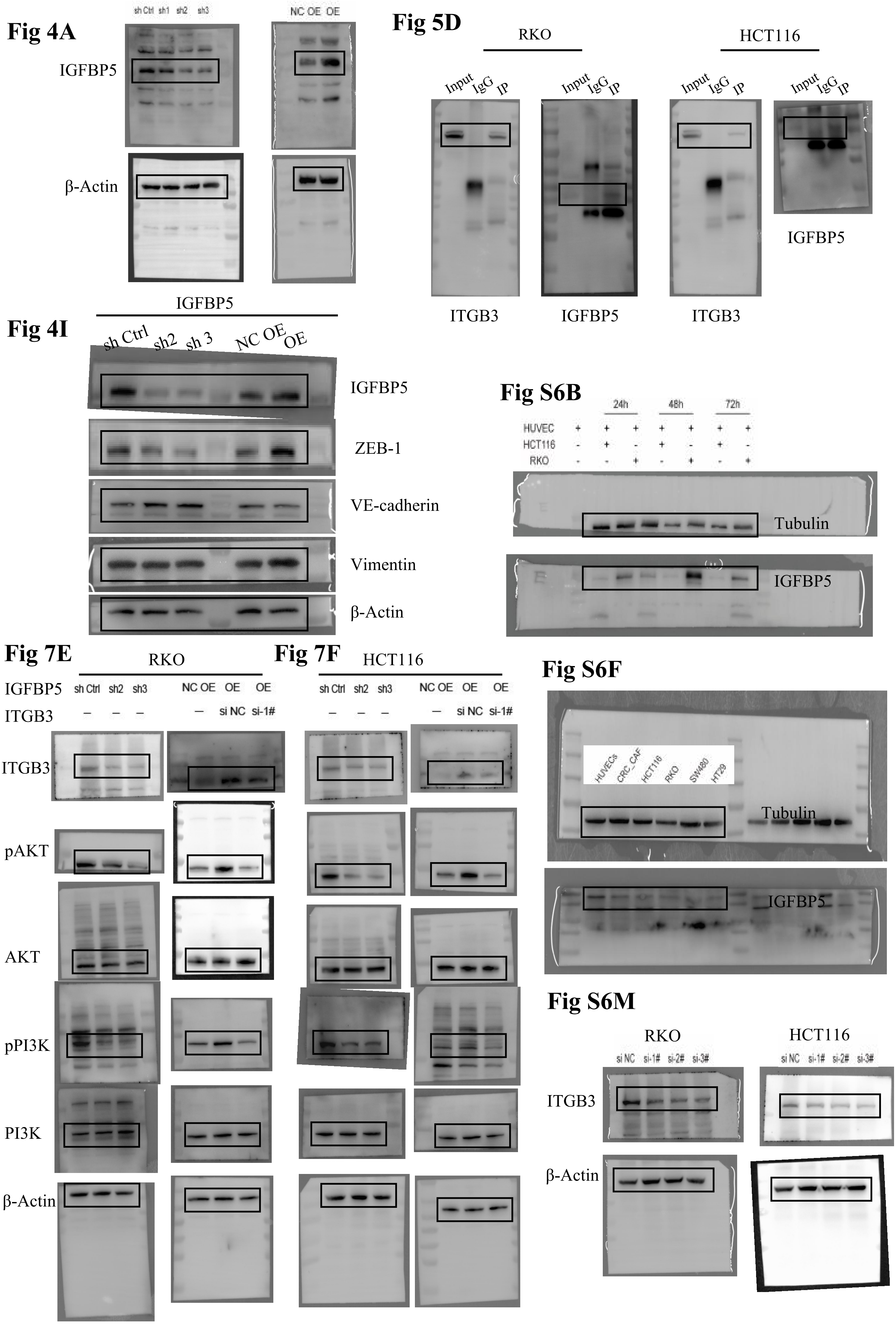
Fig 4A
sh Ctrl sh1 sh2 sh3
NC OE OE
IGFBP5
β-Actin
Fig 5D
RKO
HCT116
Input IgG IP
ITGB3
IGFBP5
ITGB3
IGFBP5
Fig 4I
IGFBP5
sh Ctrl sh2 sh 3 NC OE OE
IGFBP5
ZEB-1
VE-cadherin
Vimentin
β-Actin
Fig S6B
24h 48h 72h
HUVEC
HCT116
RKO
Tubulin
IGFBP5
Fig 7E
RKO
Fig 7F
HCT116
IGFBP5 sh Ctrl sh2 sh3 NC OE OE OE
ITGB3 — — — — si NC si-1#
ITGB3
pAKT
AKT
pPI3K
PI3K
β-Actin
Fig S6F
HUVECs CRC_CAF HCT116 RKO SW480 HT29
Tubulin
IGFBP5
Fig S6M
RKO
HCT116
si NC si-1# si-2# si-3#
ITGB3
β-Actin
