# Supplementary material for: IGFBP5 derived from endothelial cells drive vascular invasion and liver metastasis of colorectal cancer by activating the ITGB3/PI3K/AKT Axis
Source: Cell Death Dis. 2026 Jul 23;17(1):891. doi: 10.1038/s41419-026-09081-6 (PMC13680512; doi:10.1038/s41419-026-09081-6)
Supplement: Supplementary file 2 [file 41419_2026_9081_MOESM1_ESM.docx]

**Supplementary Methods**

*Quality Control and Preprocessing*

Low-quality cells were systematically excluded using stringent filtering criteria: (1) Cells expressing fewer than 3 unique genes; (2) Cells with a total gene count below 50; (3) Cells exhibiting mitochondrial gene content ≥ 20% or hemoglobin gene content ≥ 5% of total transcripts.

*Normalization, Integration, and Dimensionality Reduction*

Filtered cell data underwent normalization and variance stabilization via SCTransform, which concurrently identified highly variable genes for subsequent analysis. To address inter-sample batch effects and enable integrated analysis, data harmonization was performed using the Harmony algorithm. Principal component analysis (PCA) was executed, with dimensionality selection informed by elbow plot evaluation.

*Cell Clustering and Visualization*

Cell clusters were identified by constructing a shared nearest-neighbor graph (FindNeighbors ()) followed by community detection (FindClusters ()). Cluster relationships were visualized in two dimensions using uniform manifold approximation and projection (UMAP).

*Cellular Annotation and Subpopulation Resolution*

Cluster-defining marker genes were ascertained using FindAllMarkers (), focusing exclusively on significantly upregulated transcripts for cell type annotation. To dissect cellular heterogeneity within major populations, iterative subclustering was performed. This involved reapplying the core analytical workflow (normalization, dimensionality reduction, and clustering) to each targeted population, enabling the identification of transcriptionally distinct subtypes based on characteristic marker gene profiles. Comprehensive details regarding sample processing are provided in the Supplementary Methods section.

**Proteins extraction, Co-IP and western blotting analysis**

Total proteins were extracted using RIPA lysis buffer (P0013C, Beyotime) supplemented with Protease and phosphatase inhibitor cocktail (1:50, P1045, Beyotime) and quantified by BCA Protein Assay Kit (ZJ101, EpiZyme). Equal amounts of total proteins were separated by SDS-PAGE and transferred to PVDF membranes (0.2um, ISEQ00010, Millipore Immobilon-PSQ PVDF), which were then blocked with 5% bovine serum albumin or milk powder to block the nonspecific bindings. Then incubated with primary antibodies at 4℃ overnight, IGFBP5 polyclonal antibody (1:800, 55205-1-AP, RRID: AB_2736835, Proteintech, China), ITGB3 monoclonal antibody (1:2500, 66952-1-Ig, AB_2128759, RRID:AB_2128759, Proteintech, China), β-Actin monoclonal antibody (1:50000, 66009-1-Ig, RRID:AB_2883475, Proteintech, China), PI3 Kinase p85 antibody (1:1000, #4292, RRID:AB_329869, cell signal technology[CST]), Phospho-PI3 Kinase p85 (Tyr458) (1:1000, #4228, RRID:AB_659940, CST), Akt Antibody (1:1000, #9272, RRID:AB_329827, CST), Phospho-Akt (Ser473)( 1:1000, #4060, RRID:AB_2315049, CST). After washed three times, the membranes were incubated with HRP-conjugated secondary antibodies at room temperature and then washed three times with TBST. The immune bands were detected using an enhanced chemiluminescence kit (PK10003, Proteintech) and the images were captured using a chemiluminescence detection system. The quantification of the images was conducted by ImageJ. Original data for the Western blots are reported as WB Original Data.

**RNA extraction and qRT-PCR assays**

Total cellular RNA was extracted using TRIzol (Thermo Fisher Scientific, Waltham, MA, USA) and reverse-transcribed using the Hiscript III Reverse Transcriptase kit (Vazyme, Nanjing, China). The expression analysis of corresponding RNA in different cell types was evaluated by qRT-PCR with Hieff qPCR SYBR Green Master Mix kit (Vazyme, Nanjing, China) using specific primers for each gene. GAPDH served as the internal reference gene. Relative RNA expression levels were calculated using the 2-△△Ct method. The primer sequences used in this study are provided in **Table S5**.

**Generation and harvasted of CRC PDOs**

Human CRC tissues were obtained from patients undergoing curative surgical resection at our hospital. Written informed consent was obtained from all participants prior to sample collection. The freshly resected tumor tissues were immediately placed in PBS with 1% P/S. The samples were processed within 2 hours of resection. After washing to remove blood and debris, the tissues were minced into around 1mm³ fragments and enzymatically digested in tumor tissue digestive fluid for 30-90 minutes at 37°C with gentle agitation. The digestion was quenched with an equal volume of Advanced DMEM/F-12 and passed through a 100μm cell strainer. The filtrate was centrifuged at 300×g for 5 minutes to pellet the cells and crypt fragments, resuspended in Matrigel (M315066, BioGenous, China) and seeded into 24 well plates. Cultures were maintained in custom Human Colonic Organoid medium (K2003‐HC, BioGenous, China) at 37°C in 5% CO_2_ and monitored daily for organoid generation.PDOs were passaged once every 7 to 10 days at a 1:3 ratio.

Organoids were harvested from gel matrices by washing briefly with PBS, incubating with Organoid dissociation solution (E238001, BioGenous, China) at 37°C for up to 30 min, and pelleting at 300 × g for 5 min. The supernatants were discarded, and the cell pellets were washed with 10 mL of PBS twice. The cell pellet should be resuspended in Matrigel to be passaged or experimented according to the study design.

**Spatial Transcriptomics Data Processing**

*Data Acquisition and Preprocessing*
Four Visium samples (VLM1–VLM4) from GSE217414 were analyzed. Raw gene expression matrices were imported using Scanpy (v1.9.0). Spots without valid spatial coordinates were filtered out. Quality control metrics included mitochondrial gene percentage and total UMI counts per spot.

*Gene Signatures*

*IGFBP5⁺ EC signature*: PLVAP, GNG11, SPARC, HSPG2, RAMP2, COL4A1, HYAL2, CALCRL, COL4A2, IFI27, IGFBP7, A2M, EGFL7, CLEC14A, SLC9A3R2, RNASE1, TCIM, CRIP2, ESAM, TCF4, ENG, PECAM1, IGFBP4, NPDC1, SPRY1, TM4SF1, APP, CAVIN1, EMP1, IGFBP5.

*MC signature*: KRT8, KRT18, KRT19, CLDN4, DSP, CLDN3, EPCAM, ELF3, CD24, CLDN7, SOX4, RNF43, CST3, RRBP1, SPINT2, CKB, C19orf33, TFF3, PRAP1, GPX2, LMO7, TPM1, CAMK2N1, PERP, FXYD3, MDK, SFN, S100A14, SMIM22, ITGB3.

*VascI signature*: ACE, VEGFA, TGFBR2, ERBB2, MMP2, TP53, MMP9, EGFR, HIF1A, CTNNB1, AKT1, IL6, PTEN, CDH1, STAT3, MALAT1, PVT1, MTOR, TIAM1, GAS5, PTK2, HOTAIR, ESR1, CXCL8, CXCR4, NEAT1, BRAF, PIK3CA, H19, TGFB1.

*Signature Scoring and Spatial Smoothing*

For each spot, expression values of signature genes were z‑normalized across all spots. The raw signature score was defined as the mean z‑score of detected genes (minimum 5 genes required). Scores were then smoothed using a Gaussian kernel (σ = 1.0) over the 20 nearest spatial neighbors. Final scores were normalized to a 0-1 scale using global 5th and 95th percentiles across all samples to reduce batch effects while preserving biological variation.

Double‑Threshold Spatial Filtering

For each spot, the six nearest neighbors were determined by Euclidean distance. A spot was classified as a high‑confidence hotspot only if: (1) its own normalized score > 0.7, and (2) the mean score of its six neighbors > 0.7.

*iStar Super‑Resolution Inference*

The iStar algorithm was applied with default parameters. Briefly, this weakly supervised model performs self‑supervised pretraining on histology images using a hierarchical vision transformer, followed by weakly supervised learning to predict pixel‑level gene expression. Spot‑level expression was deconvolved into super‑pixels by integrating histological features, enabling near single‑cell resolution mapping of signature scores and ligand‑receptor colocalization.
