# Supplementary material for: IGFBP5 derived from endothelial cells drive vascular invasion and liver metastasis of colorectal cancer by activating the ITGB3/PI3K/AKT Axis
Source: Cell Death Dis. 2026 Jul 23;17(1):891. doi: 10.1038/s41419-026-09081-6 (PMC13680512; doi:10.1038/s41419-026-09081-6)
Supplement: Supplementary file 3 [file 41419_2026_9081_MOESM2_ESM.docx]

**Supplementary Figures:**

**Supplementary Fig. 1.** Clinical significance of VascI in CRC and scRNA-seq quality control. A, B, C) K-M survival curves for OS, PFI, and DSS of TCGA-CRC patients stratified by VascI status. D) Schematic workflow of scRNA-seq data processing. E, F) Quality control metrics showing gene, UMI, and mitochondrial percentage distributions before and after filtering. G, H) UMAP plots showing batch effect correction by sample and patient before and after Harmony integration.

**Supplementary Fig. 2.** Characterization of MCs in CRLM. A) UMAP of epithelial subclustering. B) Inferred CNV heatmap for MCs, NCs, and reference immune cells. C) Distribution of CNV scores for each cell group. D) irGSEA showing upregulated Hallmark pathways in MCs from VascI^+^ LM versus VascI^-^ LM. E) HGSEA showing different Hallmark pathways in MCs from VascI^+^ LM versus VascI^-^ LM.

**Supplementary Fig. S3.** Cell-cell interaction landscape in CRLM. A, B, C, D) Pseudo-three-dimensional spatial mapping of cell-cell interactions in VascI^+^ LM (A, C) and VascI^-^ LM (B, D) by CSOmap. E, F) Sankey diagrams quantifying interaction strengths between MCs and other cell types in VascI^+^ LM (E) and VascI^-^ LM (F).

**Supplementary Fig. 4.** Spatial interaction analysis of IGFBP5_tipEC in CRLM microenvironments. A, B, C, D) Pseudo-three-dimensional spatial mapping of cell-cell interactions in (A, C) VascI^+^ LM and (B, D) VascI^-^ LM samples by CSOmap. E, F) Sankey diagrams quantifying specific interaction strengths between IGFBP5_tipEC and other cell types in (E) VascI^+^ LM and (F) VascI^-^ LM microenvironments.

**Supplementary Fig. 5.** Trajectory analysis of endothelial cell differentiation in CRLM. A, B, C) Endothelial differentiation trajectories inferred by (A) Monocle, (B) Slingshot, and (C) Vector algorithms, positioning IGFBP5_tipEC at the trajectory terminus. D, E, F) CytoTRACE2 analysis validating the mature developmental status of IGFBP5_tipEC through (D) differentiation potential mapping, (E) developmental pseudotime distribution, and (F) gene count versus CytoTRACE2 score correlation.

**Supplementary Fig. 6.** IGFBP5 Derived From ECs Drive Vascular Invasion and Liver Metastasis of Colorectal Cancer by Activating the ITGB3/PI3K/AKT Axis. A) Validation of IGFBP5 knockdown and overexpression efficiency at mRNA levels by qRT-PCR (n=3). B) WB analysis of IGFBP5 protein expression in HUVECs co-cultured with HCT116 or RKO cells for different durations. C) Venn diagram showing overlap of DEGs between IGFBP5-OE and IGFBP5-sh comparisons. D) Volcano plots of differentially expressed genes in IGFBP5-sh vs NC and IGFBP5-OE vs NC ECs. E). GO and KEGG analysis of overlap of DEGs between IGFBP5-OE and IGFBP5-sh comparisons. F) WB analysis of IGFBP5 protein expression across various cell types including ECs, CAFs, and CRC cell lines. G) mIF detection of IGFBP5 expression in cells under different co-culture conditions (Scale bar = 100 μm). H) Volcano plots of differentially expressed genes in tumor cells co-cultured with IGFBP5-sh ECs vs control and IGFBP5-OE ECs vs control. I) GO and KEGG enrichment analysis of DEGs showing top enriched biological processes. J) Venn diagram showing overlap of DEGs from co-cultured tumor cells and ECs, identifying ITGB3, PROS1, and RF00019 as common targets. K) Detailed molecular docking parameters and binding affinity confirmation. L) Cellular immunofluorescence showing co-localization of IGFBP5 (green) and ITGB3 (red) in RKO and HCT116 (Scale bar = 10 μm). M, N) Validation of ITGB3 knockdown efficiency by WB (M) and qRT-PCR (N). O) GSEA plot of PI3K/AKT signaling pathway.
