# Supplementary material for: IGFBP5 derived from endothelial cells drive vascular invasion and liver metastasis of colorectal cancer by activating the ITGB3/PI3K/AKT Axis
Source: Cell Death Dis. 2026 Jul 23;17(1):891. doi: 10.1038/s41419-026-09081-6 (PMC13680512; doi:10.1038/s41419-026-09081-6)
Supplement: Supplementary file 4 [file 41419_2026_9081_MOESM3_ESM.docx]

**Supplementary Tables:**

| Table S1. Clinical characteristics, follow-up data, and IGFBP5 expression of patients from our cohort | | | | | |
| --- | --- | --- | --- | --- | --- |
| ID | Status  (1=death) | Time(month) | IGFBP5 Expression  (based on H-score) | Age | Gender (1=male) |
| 1 | 1 | 32.12 | Low | 57 | 0 |
| 2 | 0 | 84.00 | Low | 56 | 1 |
| 3 | 0 | 108.00 | Low | 43 | 0 |
| 4 | 0 | 88.00 | Low | 50 | 1 |
| 5 | 1 | 13.32 | Low | 58 | 1 |
| 6 | 0 | 83.00 | Low | 60 | 1 |
| 7 | 1 | 20.73 | Low | 70 | 1 |
| 8 | 0 | 87.00 | Low | 56 | 1 |
| 9 | 1 | 27.43 | Low | 55 | 1 |
| 10 | 0 | 91.00 | Low | 74 | 0 |
| 11 | 0 | 115.00 | Low | 62 | 0 |
| 12 | 0 | 90.00 | Low | 59 | 0 |
| 13 | 0 | 90.00 | Low | 55 | 1 |
| 14 | 1 | 32.12 | Low | 57 | 0 |
| 15 | 1 | 84.00 | Low | 56 | 1 |
| 16 | 0 | 108.00 | Low | 43 | 0 |
| 17 | 0 | 85.00 | Low | 59 | 1 |
| 18 | 0 | 88.00 | Low | 50 | 1 |
| 19 | 1 | 13.32 | Low | 58 | 1 |
| 20 | 0 | 83.00 | Low | 60 | 1 |
| 21 | 0 | 74.00 | High | 67 | 0 |
| 22 | 1 | 15.80 | High | 57 | 1 |
| 23 | 1 | 3.23 | High | 64 | 1 |
| 24 | 1 | 40.57 | High | 50 | 0 |
| 25 | 0 | 92.00 | High | 46 | 1 |
| 26 | 1 | 59.00 | High | 50 | 1 |
| 27 | 1 | 32.12 | High | 47 | 0 |
| 28 | 1 | 43.93 | High | 38 | 1 |
| 29 | 1 | 36.00 | High | 60 | 1 |
| 30 | 0 | 75.00 | High | 67 | 0 |
| 31 | 1 | 82.00 | High | 47 | 1 |
| 32 | 1 | 15.00 | High | 29 | 1 |
| 33 | 1 | 32.12 | High | 50 | 1 |
| 34 | 0 | 71.00 | High | 54 | 1 |
| 35 | 0 | 74.00 | High | 67 | 0 |
| 36 | 1 | 15.80 | High | 57 | 1 |
| 37 | 1 | 3.23 | High | 64 | 1 |
| 38 | 1 | 40.57 | High | 50 | 0 |
| 39 | 0 | 86.00 | High | 50 | 1 |
| 40 | 1 | 32.12 | High | 47 | 0 |

| ­­­­Table S2. Clinical characteristics of patients included in the single-cell RNA sequencing (scRNA-seq) analysis | | | | | | | | | | | | |
| --- | --- | --- | --- | --- | --- | --- | --- | --- | --- | --- | --- | --- |
| Age | Gender | Neoadjuvent therapy | GGT | PLT | ALB | AST | ALT | BMI | Diabetes | Hypertension | Vascular invasion |  |
| 57 | male | Yes | 212.3 | 211 | 40.3 | 56.1 | 79.1 | 22.46003435 | No | No | No |  |
| 81 | male | No | 33.6 | 407 | 36.2 | 16.3 | 14.8 | 22.14532872 | Yes | No | Yes |  |
| 55 | female | Yes | 51.6 | 216 | 40.6 | 38.8 | 24.5 | 29.76083761 | No | No | Yes |  |
| 38 | female | Yes | 15 | 165 | 39.6 | 16.3 | 18.7 | 20.93212012 | No | No | No |  |
| 50 | male | Yes | 78.2 | 169 | 41.3 | 35.6 | 36.5 | 25.79591837 | No | No | Yes |  |
| 50 | male | Yes | 82.9 | 92 | 32.3 | 27.4 | 24 | 26.0932752 | No | No | Yes |  |
| 35 | male | No | 275.8 | 348 | 43 | 50.9 | 82 | 19.72 | No | No | No |  |
| 55 | male | No | 35.8 | 215 | 35.8 | 441.7 | 371.3 | 24.8015 | No | Yes | No |  |

| Table S3. The sequences of the shRNA targeting gene. | | |  |
| --- | --- | --- | --- |
| ID | Sh | sequence | |
| 1 | IGFBP5-RNAi (sh1) | CGACGAGAAAGCCCTCTCCAT | |
| 2 | IGFBP5-RNAi (sh2) | GCAAAGGATTCTACAAGAGAA | |
| 3 | IGFBP5-RNAi (sh3) | GCAAGTCAAGATCGAGAGAGA | |
| 4 | ITGB3-RNAi (sh1) | GGCCAGAUGAUUCGAAGAATT | |
| 5 | ITGB3-RNAi (sh2) | GCCCAUGUUUGGCUACAAATT | |
| 6 | ITGB3-RNAi (sh3) | GCUCAUUGUUGAUGCUUAUTT | |

| Table S4. The sequences of the siRNA targeting ITGB3. | | |
| --- | --- | --- |
| ID | sense(5'-3') | sense(5'-3') |
| 1 | ITGB3-Homo-391 | GGCCAGAUGAUUCGAAGAATT |
| 2 | ITGB3-Homo-668 | GCCCAUGUUUGGCUACAAATT |
| 3 | ITGB3-Homo-1139 | GCUCAUUGUUGAUGCUUAUTT |

| Table S5. Primer sequences used for qRT-PCR analysis. | | |
| --- | --- | --- |
|  | name | primer sequence |
| 1 | β-actin-F | CATGTACGTTGCTATCCAGGC |
| 2 | β-actin-R | CTCCTTAATGTCACGCACGAT |
| 3 | IGFBP5-F | CCCAATTGTGACCGCAAAGG |
| 4 | IGFBP5-R | GGCAGCTTCATCCCGTACTT |
| 5 | ITGB3-F | AGTAACCTGCGGATTGGCTTC |
| 6 | ITGB3-R | GTCACCTGGTCAGTTAGCGT |
